# The regulatory mechanism of garlic skin improving the growth performance of fattening sheep through metabolism and immunity

**DOI:** 10.3389/fvets.2024.1409518

**Published:** 2024-05-30

**Authors:** Yongjie Xu, Mingliang Yi, Shixin Sun, Lei Wang, Zijun Zhang, Yinghui Ling, Hongguo Cao

**Affiliations:** ^1^College of Animal Science and Technology, Anhui Agricultural University, Hefei, China; ^2^Anhui Province Key Laboratory of Local Livestock and Poultry Genetic Resource Conservation and Bio-breeding, Anhui Agricultural University, Hefei, China

**Keywords:** GAS, fattening sheep, growth performance, metabolism, transcriptome

## Abstract

**Objective:**

Garlic skin (GAS) has been proven to improve the growth performance of fattening sheep. However, the mechanism by which GAS affects fattening sheep is not yet clear. The aim of this study is to investigate the effects of adding GAS to feed on the growth performance, rumen and fecal microbiota, serum and urine metabolism, and transcriptomics of rumen epithelial cells in fattening sheep.

**Methods:**

GAS with 80 g/kg dry matter (DM) was added to the diet of fattening sheep to study the effects of GAS on gut microbiota, serum and urine metabolism, and transcriptome of rumen epithelial tissue in fattening sheep. Twelve Hu sheep (body weights; BW, 23.0 ± 2.3 kg and ages 120 ± 3.5 d) were randomly divided into two groups. The CON group was the basal diet, while the GAS group was supplemented with GAS in the basal diet. The trial period was 10 weeks, with the first 2 weeks being the pre-trial period.

**Results:**

The daily average weight gain of fattening sheep in the GAS group was significantly higher than that in the CON group (*p* < 0.05), and the serum GSH-Px of the GAS group fattening sheep was significantly increased, while MDA was significantly reduced (*p* < 0.05). Based on the genus classification level, the addition of garlic peel in the diet changed the intestinal microbial composition, and the relative abundance was significantly upregulated by *Metanobrevibater* (*p* < 0.05), while significantly downregulated by *Akkermansia*, *Parasutterella*, and *Guggenheimella* (*p* < 0.05). Metabolomics analysis found that there were 166 significantly different metabolites in serum and 68 significantly different metabolites in urine between the GAS and CON groups (*p* < 0.05). GAS had an impact on amino acid metabolism, pyrimidine metabolism, methane metabolism, riboflavin metabolism, and unsaturated fatty acid synthesis pathways (*p* < 0.05). Transcriptome sequencing showed that differentially expressed genes were mainly enriched in immune regulatory function, improving the health of fattening sheep.

**Conclusion:**

Adding GAS can improve the energy metabolism and immune function of fattening sheep by altering gut microbiota, metabolome, and transcriptome, thereby improving the growth performance of fattening sheep.

## 1 Introduction

Garlic, as an herbaceous plant, is a natural broad-spectrum plant-based antibiotic due to its unique organic sulfur compounds, which have functions such as antibacterial, anti-inflammatory, antioxidant, and immune regulation (1, 2). Additionally, garlic is rich in vitamins and amino acids, making it a good nutritional supplement (3, 4). Garlic skin (GAS), as a by-product of garlic processing, has similar detoxification, sterilization, disease prevention, and treatment effects as garlic (5). In addition, GAS also contains abundant vitamins and minerals, which are of great significance for enhancing animal physical fitness and improving production performance (6). Research has found that GAS contains allicin, which can increase gastric secretion and gastrointestinal peristalsis, stimulate appetite and promote digestion, kill various pathogenic microorganisms such as *Staphylococcus*, *Bacillus*, and *Salmonella*, prevent and treat diseases such as enteritis, dysentery, and coccidiosis, thereby improving livestock production performance and reducing production costs (7–10). In addition, allicin has antioxidant ability, which can inhibit the production of ROS, reduce animal energy consumption, and improve production efficiency (11, 12).

Healthy breeding is the current direction of animal husbandry development, which not only solves the environmental pollution problem of animal husbandry, but also improves the health level of animals, and promotes the healthy and sustainable development of modern animal husbandry (13, 14). In the development of traditional animal husbandry, antibiotics or related drugs are widely used to reduce the occurrence of animal diseases and improve animal production performance (15). However, the abuse of antibiotics, such as over dosage and over range use, has posed a serious threat to quality of animal products and food safety (16). With the reduction and prohibition of antibiotic use in animal husbandry, research on antibiotic substitutes has become an important research direction in modern animal husbandry (17, 18). Therefore, the application of GAS as a substitute for antibiotics in the sheep farming industry can reduce economic costs and the threat caused by drug-resistant bacteria, which is of great significance for achieving healthy sheep farming. However, there is still limited research on the application of GAS in healthy breeding of meat sheep. This study added GAS to the feed of fattening sheep to explain the regulatory mechanism of GAS on the growth performance of fattening sheep from multiple aspects such as rumen and fecal microbiota, serum and urine metabolism, and rumen epithelial cell transcriptomics. This provides a theoretical basis for the promotion and utilization of GAS as a substitute for antibiotic additives in animal feed, improvinging animal growth performance.

## 2 Materials and methods

### 2.1 Experimental animals and experimental design

**Figure 1:**
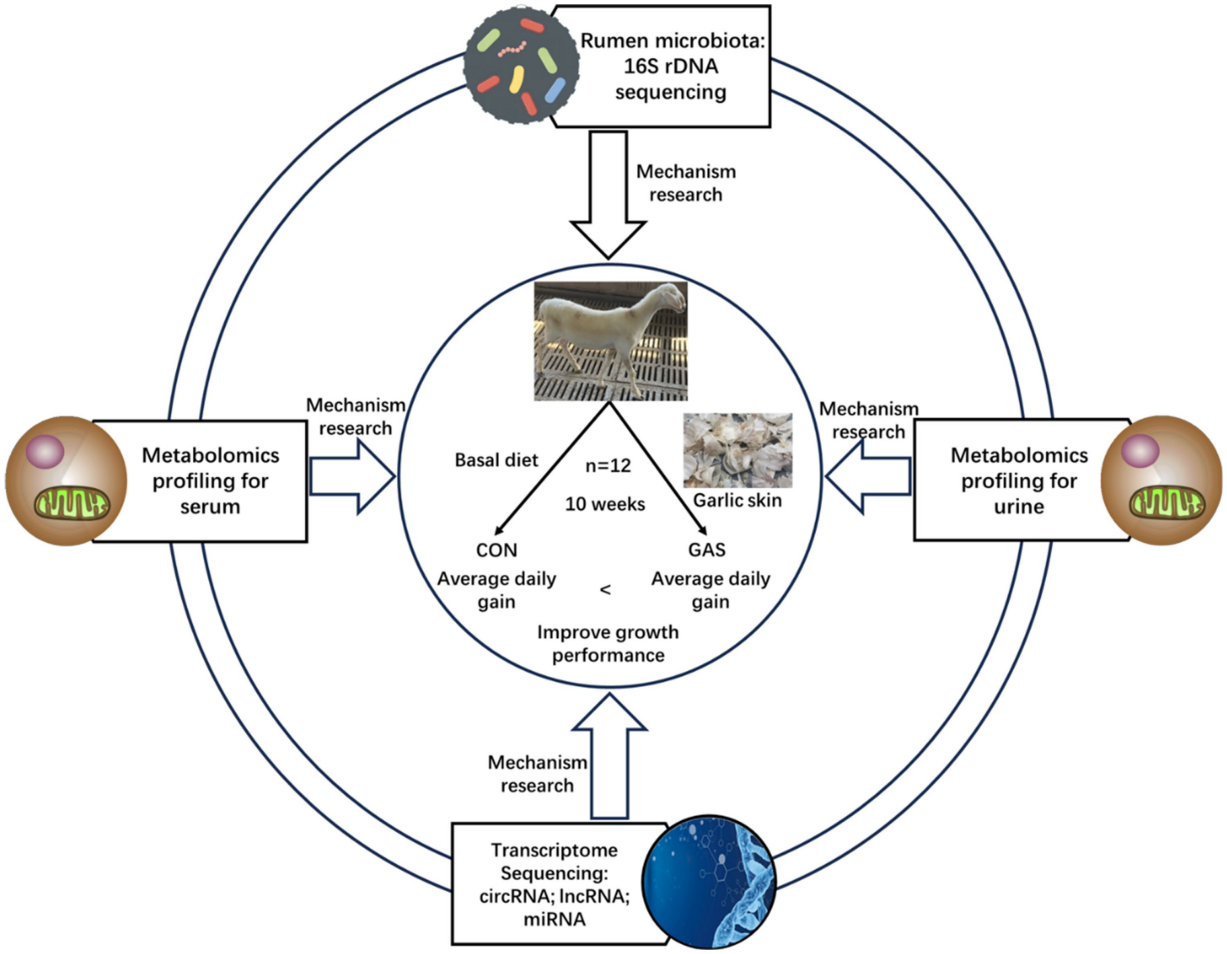
Experimental design and workflow of feeding fattening lamb with garlic skin diet. Including fecal microbiome, serum and urine metabolome, and transcriptome sequencing of rumen epithelial cells. Twelve Hu lamb were randomly assigned to a basal diet (CON) or a basal diet supplemented with 80 g/kg garlic skin DM (GAS).

This experiment was conducted on the basis of adding 80 g/kg dry matter (DM) garlic skin (GAS) to the feed of fattening sheep to improve their growth performance (19). Twelve healthy 3.5 month old Hu sheep (Chinese native sheep breeds) with similar weight (23.0 ± 2.3 kg) were selected and randomly divided into two groups, with 6 sheep in each group. The control group (CON) was fed a basal diet, while the GAS group (GAS) was fed a basal diet supplemented with 80 g/kg DM GAS, with free eating and drinking water. The experimental period was 10 weeks, with a pre-feeding period of 2 weeks and a regular feeding period of 8 weeks. Before the experiment began, the enclosure was disinfected and routine epidemic prevention measures such as deworming were taken on the experimental sheep. During the experiment, the sheep shed maintained ventilation, suitable temperature, and humidity. During the experiment, the nutritional composition indicators of the CON group basal diet and the GAS group 8% GAS diet for fattening sheep are shown in Table 1 (19). The experimental design and workflow are shown in the Figure 1.

**Table 1:** Main components of the diet for fattening lamb during the experimental period.

Item	CON	GAS
Ingredient (%)		
Ground corn grain	28.00	25.76
Soybean meal	15.00	13.80
Rapeseed Meal	9.00	8.28
Wheat bran	4.00	3.68
Sodium bicarbonate	1.00	0.92
Salt	1.00	0.92
Dicalcium phosphate	0.50	0.46
Calcium carbonate	0.50	0.46
Premix^1^	1.00	0.92
Garlic skin	0.00	8.00
Peanut straw	15.00	13.80
Soybean straw	25.00	23.00
Chemical composition (%, DM)		
Organic matter	91.30	91.40
CP	15.10	14.49
NDF	38.70	39.62
ADF	23.20	25.31
Ether extract	3.10	2.96
Calcium	0.75	0.74
Phosphorus	0.43	0.42
Metabolizable energy2, MJ/Kg	9.83	9.74

CON, control diet; GAS, garlic skin diet. ^1^Formulated to provide (per kilogram of premix) 600 KIU of Vitamin A, 80 KIU of vitamin D3, 5,000 IU of Vitamin E, 8000 mg of Zn, 60 mg of Se, 200 mg of I, 9400 mg of Fe, 72 mg of Co, 10,400 mg of Mn, and 1,600 mg of Cu.

### 2.2 Collection and determination of samples

Before feeding on the last morning of the experiment, feces, serum, and urine samples were collected from fattening sheep. Feces were collected using rectal sampling method and placed in a 2 mL cryotube in liquid nitrogen for fecal microbiological analysis. Blood was collected from the fattening sheep using an empty stomach jugular vein blood collection method, which was left at room temperature for 2 h, centrifuged at 3500 r/min for 15 min, and the supernatant was serum, after absorbing the serum, it was divided into 2 mL cryotubes and stored in liquid nitrogen. Fasting fattening sheep blood was collected using the jugular vein blood collection method. It was left to stand at room temperature for 2 h, centrifuged at 3500 r/min for 15 min, and the supernatant serum was absorbed. The serum was then loaded into a 2 mL cryotube and placed in liquid nitrogen, with a portion of the serum used for serum antioxidant index analysis, including total antioxidant capacity (T-AOC) (A015-1-2, Nanjing Institute of Biotechnology), superoxide dismutase (SOD) (A001-3-2, Nanjing Institute of Biotechnology) Glutathione peroxidase (GSH-Px) (A005-1-2, Nanjing Jiancheng Institute of Biotechnology) and catalase (CAT) activity (A007-1-1, Nanjing Jiancheng Institute of Biotechnology), as well as malondialdehyde (MDA) content (A003-1-2, Nanjing Jiancheng Institute of Biotechnology). The remaining amount was used for serum metabolomics analysis. Fresh urine from 12 fattening sheep was collected and stored in a 2 mL cryotube in liquid nitrogen for metabolomics analysis of urine.

At the end of the experiment, fasting fattening sheep were slaughtered, and rumen epithelial tissue was collected. The tissue was rinsed with physiological saline and stored in a 2 mL cryotube and liquid nitrogen for transcriptome sequencing. The collected gastrointestinal tissue was fixed with 4% paraformaldehyde, and histological sections were made using HE staining method. Neutral gum was used for sealing and microscopy observation. All experimental operations were carried out wearing disposable sterile masks and gloves to avoid contamination.

### 2.3 16S rRNA sequencing of fecal microorganisms

The fecal samples were thawed at 4°C, and the genomic DNA of the fecal samples was extracted using E.Z.N.A. fecal DNA kit (Omega Bio tek, Norcross, GA, United States). The DNA samples were measured at OD260/OD280 ratio, and the DNA integrity was detected by 1% agarose gel electrophoresis. Using DNA as a template, the PCR amplification was performed by using specific primers for the variable region of 16S rRNA V3-V4. After passing the PCR product detection and purification, it was sent to Shanghai Shenggong Biotechnology Services Co., Ltd. for high-throughput 16S 156 rRNA sequencing of fecal microorganisms based on the Illumina Miseq platform.

### 2.4 Metabolomics detection of serum and urine

Serum and urine samples were sent to Shanghai Baiqu Biotechnology Co., Ltd. for metabolomics testing and analysis. The entire experimental process includes sample processing, non-targeted metabolomics testing, and analysis, all completed by Shanghai Baiqu Biotechnology Co., Ltd. A metabolomics analysis was conducted on frozen serum and urine samples of fattening sheep using Ultra-high-performance liquid chromatography-tandem quadrupole time-of-flight mass spectrometry (UPLC-Q-TOF-MS). The obtained raw data was processed and analyzed. When the *p*-value of Student’s *t*-test was less than 0.05 and the Variable Importance in the Projection (VIP) of the first principal component of the OPLS-DA model was greater than 1, it was considered that there was a significant difference in metabolites. Subsequently, metabolic pathway analysis of differential metabolites was conducted.

### 2.5 Transcriptomics analysis

The frozen fattening sheep rumen epithelial tissue samples were transported to Shanghai Sangong Biotechnology Co., Ltd. with dry ice for transcriptome sequencing. First, the total RNA was extracted from the samples by the Trizol method for purity and concentration detection, and the integrity of RNA was detected by 1% agarose gel electrophoresis. The qualified samples were sequenced using Illumina HiSeqTM high-throughput sequencing platform, and the sequencing data were processed and analyzed. Using DESeq2 for differential expression gene analysis, the screening conditions were: *p*-value<0.05 and fold of difference | Fold Change | > 2. Annotate the differential gene expression function and explore the biological functions involved in differential expression genes (DEGs), mainly including biological processes, cellular components, and molecular functions. Cluster Profiler was used for functional enrichment analysis, and when *p*-value<0.05, this function was enriched.

### 2.6 Statistical analysis

All experimental data were organized using Excel, and the SPSSAU data analysis platform^1^ was used for the normal distribution test of the data. The SPSS software independent sample *T*-test was used for statistical analysis. When *p* < 0.05, the difference was significant, and *p* < 0.01, the difference was extremely significant. Each group of experiments had 6 replicates.

## 3 Results

### 3.1 Growth performance

Previous studies have found that adding 8% GAS to the diet could significantly increase the average daily feed intake and weight gain of fattening sheep (*p* < 0.05), and the feed conversion rate (average daily weight gain/DM intake) had an upward trend (0.05 < *p* < 0.10), indicating that GAS could improve the growth performance of fattening sheep (19). Based on the findings of this study, further analysis was conducted on fecal microbiota, urine and serum metabolomics, as well as rumen epithelial cell metabolomics. The mechanism of the effect of adding GAS to the diet on the growth performance of fattening sheep was thoroughly analyzed.

### 3.2 Fecal microbiota

**Figure 2:**
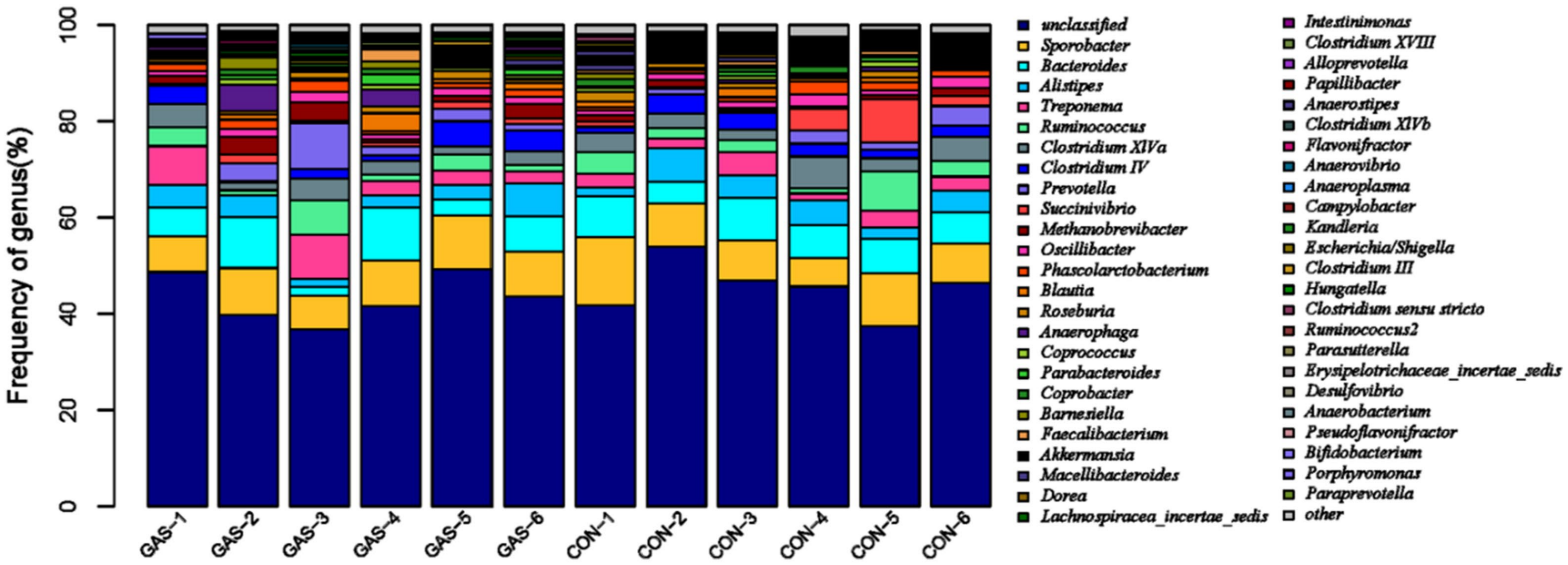
Distribution characteristics of fecal microbiota in the GAS group.

Microorganisms that coevolve in the gut of animal hosts can form a complex microecosystem within the animal organism, known as gut microbiota (20). The gut microbiota of livestock and poultry mainly consists of *Firmicutes* and *Bacteroidetes*, followed by *Proteobacteria*, *Actinobacteria*, and *Fusobacteria*. In the bar chart of the genus level community structure distribution of samples in the GAS and CON groups, the community structure of fecal microorganisms at the genus level was presented (Figure 2). *Sporobacter*, *Bacteroides*, *Alistipes*, *Treponema*, *Ruminococcus* and other bacterial genera were detected in both GAS and CON groups. Four significant differences were identified between the GAS group and the CON group, namely *Metanobrevibater*, *Akkermansia*, *Parasutterella*, and *Guggenheimella* (Table 2). Compared to the CON group, the GAS group significantly upregulated the genus *Metanobrevibate*; The significantly downregulated ones were *Akkermansia*, *Parasutterella*, and *Guggenheimella*.

**Table 2:** Change characteristics of the main microbial communities in the feces of the GAS diet group.

Phylum	Genus	Treament		SEM	*p*-value
		CON	GAS		
Euryarchaeota	Methanobrevibacter	1.042^b^	2.350^a^	0.491	0.018
Verrucomicrobia	Akkermansia	0.882^a^	0.080^b^	0.339	0.032
Firmicutes	Parasutterella	0.148^a^	0.087^b^	0.025	0.024
	Guggenheimella	0.055^a^	0.018^b^	0.017	0.046

^a,b^Values within a row with different superscripts differ significantly at *p* < 0.05.

### 3.3 Serum antioxidant and metabolomics

The level of serum antioxidant capacity reflects the level of antioxidant stress in the body, which is closely related to the health level of the body and is of great significance for the growth and development of fattening sheep. Serum antioxidant indicators were tested on 12 fattening sheep in the GAS and CON groups, including glutathione peroxidase (GSH-Px), malondialdehyde (MDA), superoxide dismutase (SOD), and total antioxidant capacity (T-AOC) (Table 3). Compared with the CON group, the GAS group significantly increased serum GSH-Px and significantly decreased MDA in fattening lamb (*p* < 0.05); SOD and T-AOC were slightly higher than those in the CON group.

**Table 3:** Effect of GAS diet on serum antioxidant capacity of fattening lamb.

Item	Treatment		SEM	*p*-value
	CON	GAS		
GSH-Px	287.419^b^	338.226^a^	17.417	0.011
MDA	2.970^a^	2.436^b^	0.498	0.049
SOD	15.551^a^	15.918^a^	1.442	0.242
T-AOC	0.288^a^	0.411^a^	0.091	0.197

^a,b^Values within a row with different superscripts differ significantly at *p* < 0.05.

**Figure 3:**
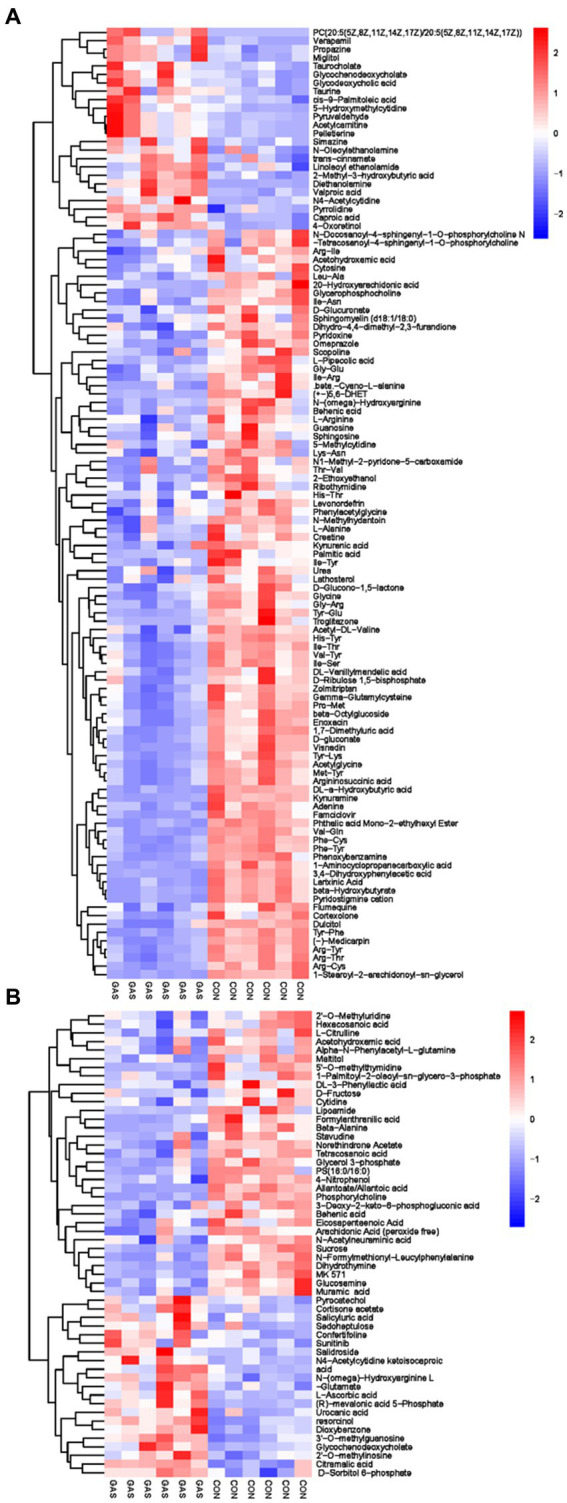
Hierarchical cluster analysis of Serum metabolites in Fattening Lamb. **(A)** Hierarchical cluster analysis of GAS group serum in cationic mode. **(B)** Hierarchical cluster analysis of GAS group serum in anionic mode.

The metabolites in serum come from various metabolic pathways of the body. Studying metabolites in serum can provide a comprehensive understanding of the body’s metabolism and evaluate its physiological and pathological status. We have screened a total of 166 differential metabolites, including 54 anionic modes and 112 cationic modes (Supplementary Table S1; Figure 3). There were significant differences in serum differential metabolites between the GAS group and the CON group in fattening sheep. Compared to the CON group, the GAS group significantly upregulated a total of 45 differential metabolites, such as Pyrocatechol, Cortisone acetate, Salicyluric acid, Verapamil, Propazine, and Miglitol, A total of 121 types of significant downregulation were observed, such as 2 ‘- O-methylinosine, Hexacosanoic acid, L-Citrulline, Acetohydroxamic acid, Cytosine, and Glycerophosphocholine.

**Figure 4:**
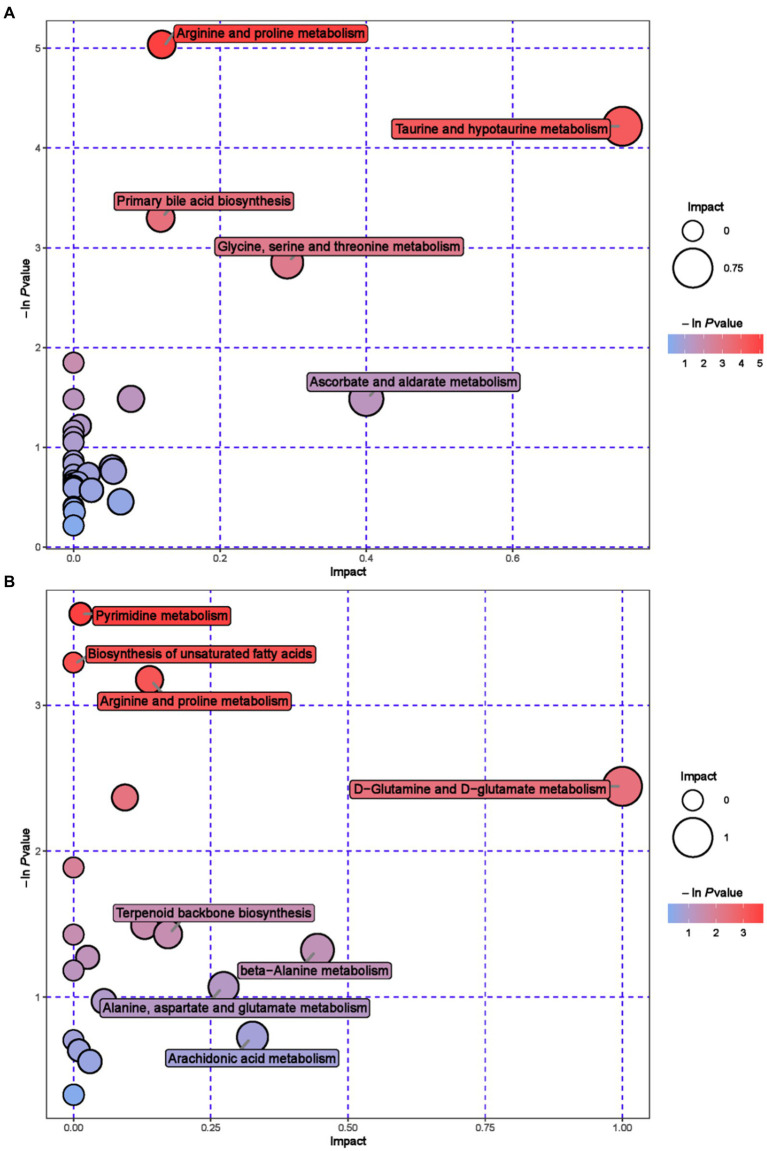
Pathway analysis of serum metabolomics. **(A)** Pathway analysis of GAS group serum in cationic mode. **(B)** Pathway analysis of GAS group serum under anionic mode.

Further metabolic pathway analysis was conducted on differential metabolites to identify the key metabolic pathways with the highest correlation with metabolic differences. We screened 42 key pathways with the highest differential correlation with metabolites between the GAS and CON groups’ serum (Supplementary Table S2; Figure 4). The key pathways with the highest differential correlation with metabolites between the GAS and CON groups were Taurine and hydroxylamine metabolism, D-glutamine and D-glutamite metabolism, Arginine and proline metabolism, Pyrimidine metabolism, Biosynthesis of unsaturated fatty acids, and Primary bill acid biosynthesis.

### 3.4 Urine metabolomics

**Figure 5:**
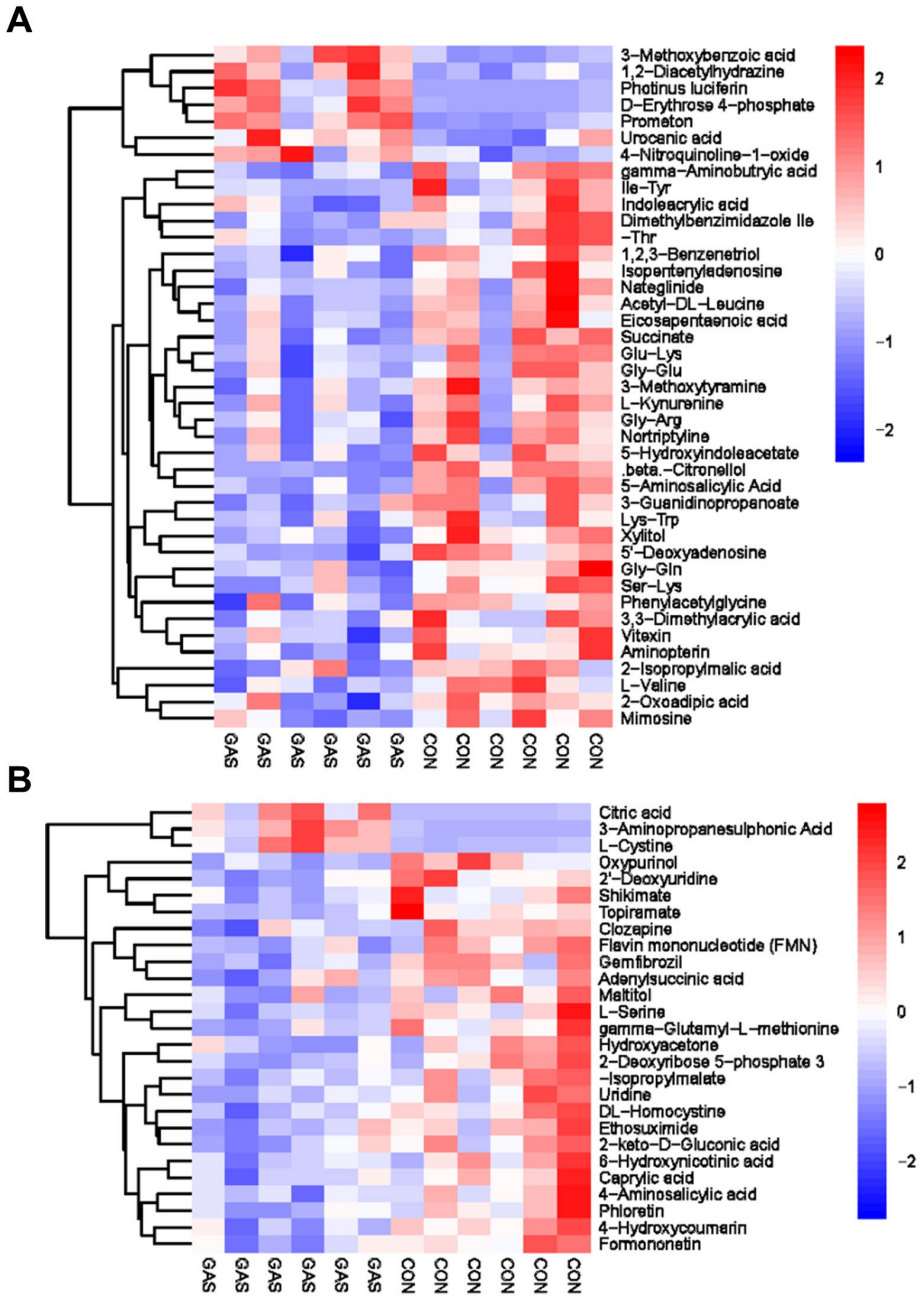
Hierarchical cluster analysis of urine metabolites in fattening lamb. **(A)** Hierarchical cluster analysis of GAS group urine in cationic mode. **(B)** Hierarchical. Cluster analysis of GAS group urine in anionic mode.

Metabolomics analysis was conducted on the urine of fattening sheep in the GAS and CON groups, identifying 68 differential metabolites in the urine, including 27 anionic modes and 41 cationic modes (Supplementary Table S3; Figure 5). The differential metabolites mainly included amino acids, fatty acids, purine pyrimidines, and organic acids. These differential metabolites were mainly divided into two categories: one was 58 significantly downregulated differential metabolites compared to the CON group, mainly including oxypurinol, 2 ‘- Deoxyuridine, Shikimate, Gamma aminobutyric acid, Indoleacrylic acid, and Dimethylbenzimidazole; The other type was significantly upregulated by 10 metabolic markers, such as Citric acid, 3-Aminopropoanesulfonic acid, L-Cystine, 3-Methodybenzoicacid, 1,2-Diacetylhydrazine, and Photinus luciferin.

**Figure 6:**
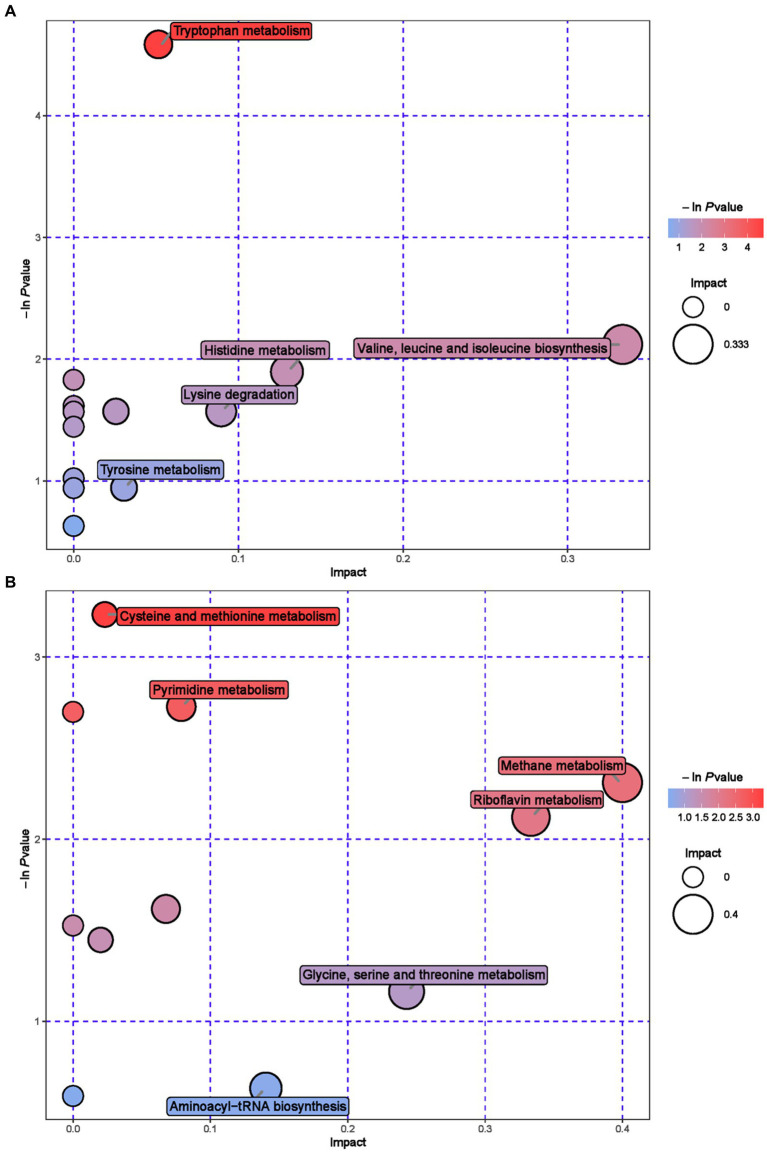
Pathway analysis of urine metabolomics. **(A)** Pathway analysis of MHB group urine in cationic mode. **(B)** Pathway analysis of MHB group urine under anionic mode.

Enrichment analysis and topological analysis were conducted on the pathways involved in differential metabolites, and 23 metabolic pathways related to differential metabolites in urine were identified (Supplementary Table S4; Figure 6). Metabolic pathway analysis showed that Cysteine and methionine metabolism, Pyrimidine metabolism, Methane metabolism, Riboflavin metabolism, Tryptophan metabolism and Valine, leucine and isoleucine biosynthesis, etc.

### 3.5 CircRNA bioinformatics

**Figure 7:**
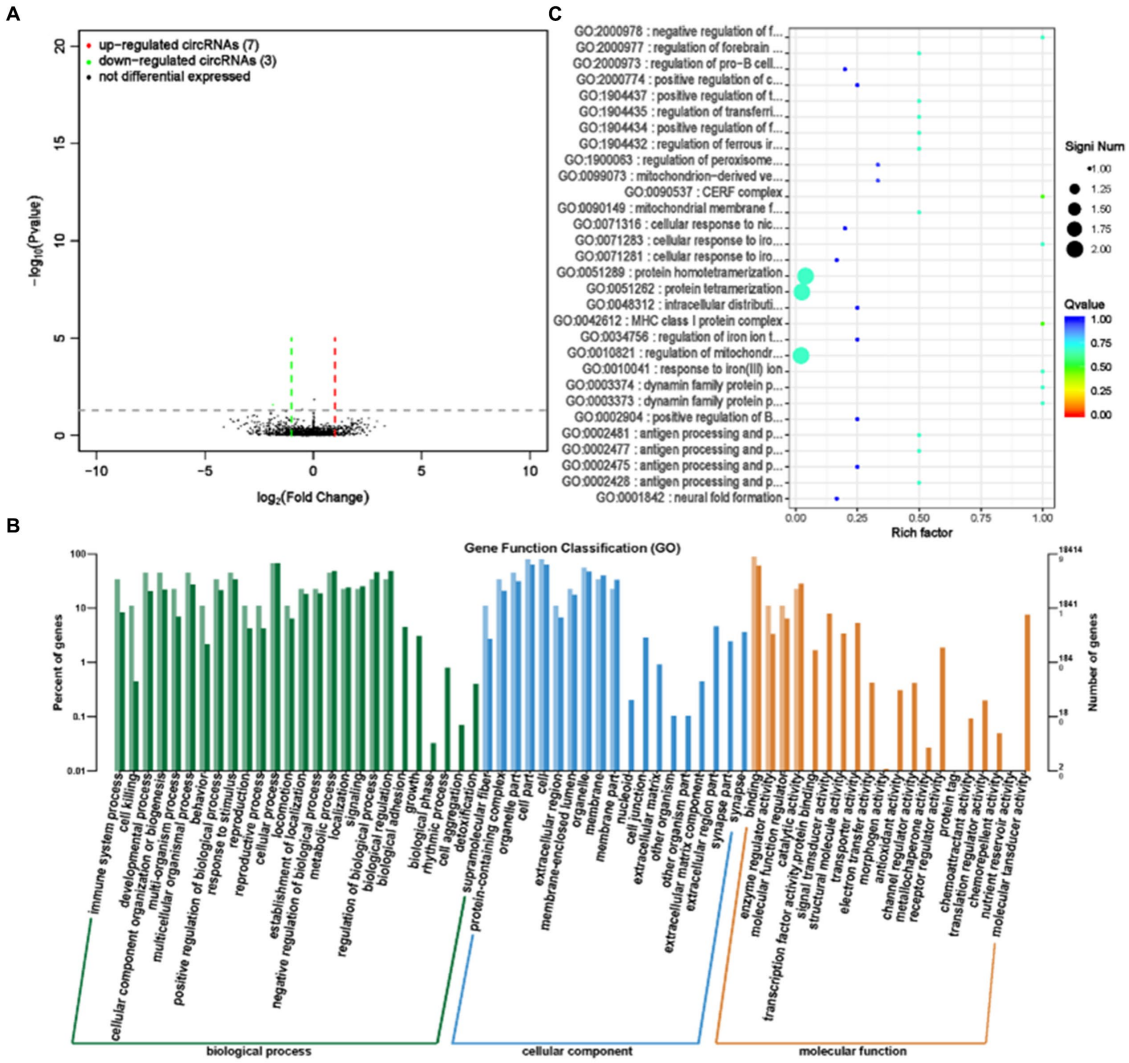
Differential expression analysis of circRNA between GAS group and CON group. **(A)** Volcano map of circRNA expression differences between GAS group and CON group. The horizontal axis represents the fold change (log (B/A)) value of the transcript expression difference between different groups, while the vertical axis represents the *P*-value of the transcript expression change. The smaller the *p*-value, the greater the – log (*p*-value), and the more significant the difference. Red represents upregulated transcripts, green represents downregulated transcripts, and black represents non differential transcripts. **(B)** Histogram of host gene functional annotation classification for differentially expressed circRNA between GAS and CON groups. The horizontal axis represents the functional classification, while the vertical axis represents the number of genes within the classification (right) and their percentage in the total number of annotated genes (left). Light colors represent host genes, while dark colors represent all genes. **(C)** The top 30 functional scatter plots show significant enrichment of circRNA between the GAS group and the CON group. The vertical axis represents functional annotation information, while the horizontal axis represents the Rich factor corresponding to the function. The size of the Q-value is represented by the color of the dot. The smaller the Q-value, the closer the color is to red. The number of differentially expressed circRNA host genes is represented by the size of the dot.

CircRNA (Circular RNA) has the characteristics of being abundant, evolutionarily conserved, and relatively stable in the cytoplasm, which enables it to have many functions and participate in gene expression regulation through various ways of action (21). We performed circRNA sequencing on rumen epithelial cell samples from the GAS and CON groups. By analyzing the differential expression of circRNA, we screened for significant differences in circRNA expression between the GAS group and the CON group. Among them, there were 10 significantly expressed differentially expressed circRNAs, of which 7 were significantly upregulated and 3 were significantly downregulated (Supplementary Table S5; Figure 7A).

After selecting differentially expressed circRNAs, GO analysis was conducted to investigate the distribution of differentially expressed circRNA target genes in annotation function between the GAS and CON groups, in order to elucidate the effect of feeding garlic skin containing diets on gene function. The target genes for differentially expressed circRNA are mainly involved in cellular processes, metabolic processes, biological regulation processes, and biological processes such as cell composition, tissue, or biogenesis. They were mainly located in the cellular and organelle parts, and their main molecular functions are focused on binding and catalytic activity (Figure 7B). To further test whether the target genes of differentially expressed circRNA are enriched in certain functions, we conducted functional enrichment analysis on the genes and selected the top 30 functions with the highest functional enrichment of differentially expressed circRNA target genes (Table 4; Figure 7C). The target genes for differentially expressed circRNA between the GAS and CON groups were mainly involved in the regulation of iron ions and immune responses, which is closely related to the efficacy and role of GAS itself. GAS contains a large amount of sulfur compounds, which can enhance the body’s immune system and effectively block the formation of lipid peroxidation (22). This is consistent with the above results.

**Table 4:** 30 significantly enriched differential circRNAs in the GAS diet group.

GO.ID^1^	Term^2^	Ontology^3^	Significant^4^	Annotated^5^	*p*-value
GO:2000978	Negative regulation of forebrain neuron differentiation	Biological process	1/6	1/14620	<0.01
GO:2000977	Regulation of forebrain neuron differentiation	Biological process	1/6	2/14620	0.001
GO:2000973	Regulation of pro-B cell differentiation	Biological process	1/6	5/14620	0.002
GO:2000774	Positive regulation of cellular senescence	Biological process	1/6	4/14620	0.002
GO:1904437	Positive regulation of transferrin receptor binding	Biological process	1/6	2/14620	0.001
GO:1904435	Regulation of transferrin receptor binding	Biological process	1/6	2/14620	0.001
GO:1904434	Positive regulation of ferrous iron binding	Biological process	1/6	2/14620	0.001
GO:1904432	Regulation of ferrous iron binding	Biological process	1/6	2/14620	0.001
GO:1900063	Regulation of peroxisome organization	biological process	1/6	3/14620	0.001
GO:0099073	Mitochondrion-derived vesicle	Cellular component	1/8	3/14551	0.002
GO:0090537	CERF complex	Cellular component	1/8	1/14551	0.001
GO:0090149	Mitochondrial membrane fission	Biological process	1/6	2/14620	0.001
GO:0071316	Cellular response to nicotine	Biological process	1/6	5/14620	0.002
GO:0071283	Cellular response to iron (III) ion	Biological process	1/6	1/14620	<0.01
GO:0071281	Cellular response to iron ion	Biological process	1/6	6/14620	0.002
GO:0051289	Protein homotetramerization	Biological process	2/6	51/14620	<0.01
GO:0051262	Protein tetramerization	Biological process	2/6	83/14620	<0.01
GO:0048312	Intracellular distribution of mitochondria	Biological process	1/6	4/14620	0.002
GO:0042612	MHC class I protein complex	Cellular component	1/8	1/14551	0.001
GO:0034756	Regulation of iron ion transport	Biological process	1/6	4/14620	0.002
GO:0010821	Regulation of mitochondrion organization	Biological process	2/6	96/14620	0.001
GO:0010041	Response to iron (III) ion	Biological process	1/6	1/14620	<0.01
GO:0003374	Dynamin family protein polymerization involved in mitochondrial fission	Biological process	1/6	1/14620	<0.01
GO:0003373	Dynamin family protein polymerization involved in membrane fission	Biological process	1/6	1/14620	<0.01
GO:0002904	Positive regulation of B cell apoptotic process	Biological process	1/6	4/14620	0.002
GO:0002481	Antigen processing and presentation of exogenous protein antigen via MHC class Ib, TAP-dependent	Biological process	1/6	2/14620	0.001
GO:0002477	Antigen processing and presentation of exogenous peptide antigen via MHC class Ib	Biological process	1/6	2/14620	0.001
GO:0002475	Antigen processing and presentation via MHC class Ib	Biological process	1/6	4/14620	0.002
GO:0002428	Antigen processing and presentation of peptide antigen via MHC class Ib	Biological process	1/6	2/14620	0.001
GO:0001842	Neural fold formation	Biological process	1/6	6/14620	0.002

^1^GO.ID: Number and name of GO. ^2^Term: Description information of GO function. ^3^Ontology: Category of GO (cellular component; biological process; molecular function).^4^Significant: Number of differentially expressed circRNA host genes annotated to the GO/Number of differentially expressed circRNA host genes annotated to the GO database. ^5^Annotated Number of genes annotated to the GO/Number of genes annotated to the GO database.

### 3.6 LncRNA bioinformatics

**Figure 8:**
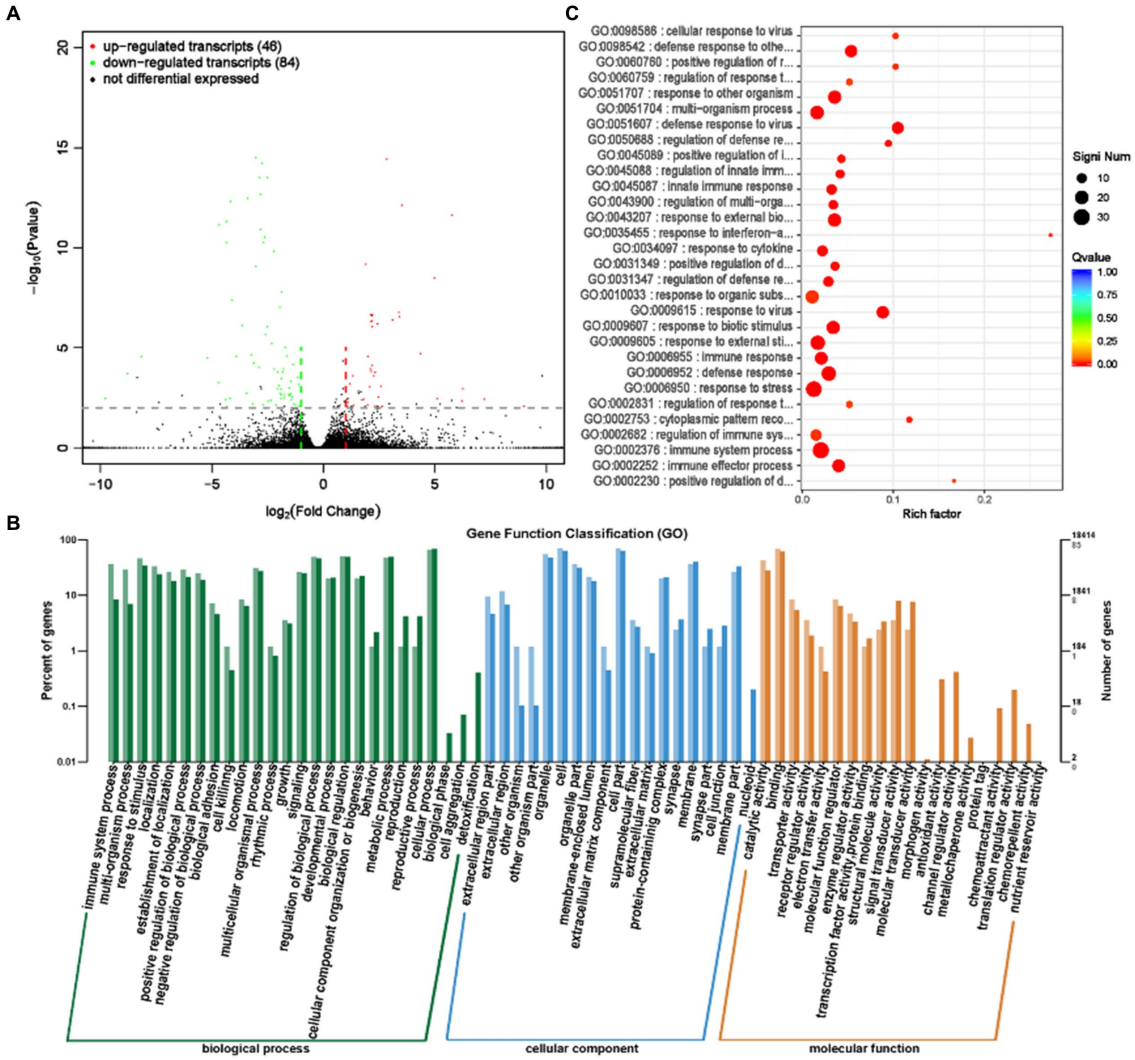
Differential expression analysis of transcriptome between GAS group and CON group. **(A)** Volcano map of transcript expression differences between GAS group and CON group. **(B)** Histogram of functional annotation classification of genes corresponding to GAS and CON differential transcripts. **(C)** The top 30 functional scatter plots show significant enrichment of differentially expressed transcripts in the GAS and CON groups.

LncRNA (Long Chain Non coding RNA) has a conserved secondary structure that can interact with proteins, DNA, and RNA, participate in the regulation of various biological processes, and play an important role in numerous life processes (23, 24). Differential analysis of transcript expression was performed on the rumen epithelial tissues of the GAS and CON groups. A total of 130 differentially expressed transcripts (lncRNA and mRNA) were screened (Supplementary Table S6; Figure 8A), with 46 significantly upregulated transcripts, including 7 significantly upregulated lncRNA, 39 mRNA, and 84 significantly downregulated transcripts, including 39 lncRNA and 45 mRNA.

The annotation analysis of gene functions corresponding to differential transcripts revealed that the gene functions of differential transcripts are mainly involved in biological processes such as cellular processes, metabolic processes, and regulation of biological processes, located in positions such as cells, cell components, and organelles (Figure 8B). The main molecular functions are concentrated in binding and catalytic activities. Functional enrichment analysis examined the enrichment function of differentially expressed genes, and the corresponding gene functions of transcripts were mainly enriched in the immune regulatory response of the body (Table 5; Figure 8C). The allicin contained in GAS has bactericidal effects, which can enhance the immunity of livestock and poultry (25). This is consistent with the analysis results of gene function enrichment corresponding to transcripts.

**Table 5:** Gene functions corresponding to 30 significantly enriched differential transcripts in the GAS group.

GO.ID	Term	Ontology	Significant	Annotated	*p*-value
GO:0098586	Cellular response to virus	Biological process	4/72	39/14620	<0.01
GO:0098542	Defense response to other organism	Biological process	16/72	298/14620	<0.01
GO:0060760	Positive regulation of response to cytokine stimulus	Biological process	4/72	39/14620	<0.01
GO:0060759	Regulation of response to cytokine stimulus	Biological process	5/72	97/14620	<0.01
GO:0051707	Response to other organism	Biological process	21/72	533/14620	<0.01
GO:0051704	Multi-organism process	Biological process	24/72	1281/14620	<0.01
GO:0051607	Defense response to virus	Biological process	15/72	143/14620	<0.01
GO:0050688	Regulation of defense response to virus	Biological process	5/72	53/14620	<0.01
GO:0045089	Positive regulation of innate immune response	Biological process	7/72	163/14620	<0.01
GO:0045088	Regulation of innate immune response	Biological process	8/72	193/14620	<0.01
GO:0045087	Innate immune response	Biological process	11/72	342/14620	<0.01
GO:0043900	Regulation of multi-organism process	Biological process	9/72	264/14620	<0.01
GO:0043207	Response to external biotic stimulus	Biological process	21/72	535/14620	<0.01
GO:0042825	TAP complex	Cellular component	2/72	2/14551	<0.01
GO:0035455	Response to interferon-alpha	Biological process	3/72	11/14620	<0.01
GO:0034097	Response to cytokine	Biological process	13/72	541/14620	<0.01
GO:0031349	Positive regulation of defense response	Biological process	9/72	223/14620	<0.01
GO:0031347	Regulation of defense response	Biological process	12/72	383/14620	<0.01
GO:0010033	Response to organic substance	Biological process	21/72	1730/14620	<0.01
GO:0009615	Response to virus	Biological process	17/72	192/14620	<0.01
GO:0009607	Response to biotic stimulus	Biological process	21/72	559/14620	<0.01
GO:0009605	Response to external stimulus	Biological process	25/72	1351/14620	<0.01
GO:0006955	Immune response	Biological process	18/72	856/14620	<0.01
GO:0006952	Defense response	Biological process	25/72	819/14620	<0.01
GO:0006950	Response to stress	Biological process	30/72	2196/14620	<0.01
GO:0002831	Regulation of response to biotic stimulus	Biological process	5/72	97/14620	<0.01
GO:0002753	Cytoplasmic pattern recognition receptor signaling pathway	Biological process	4/72	34/14620	<0.01
GO:0002376	Immune system process	Biological process	31/72	1536/14620	<0.01
GO:0002252	Immune effector process	Biological process	18/72	449/14620	<0.01
GO:0002230	Positive regulation of defense response to virus by host	Biological process	3/72	18/14620	<0.01

### 3.7 MiRNA bioinformatics

**Figure 9:**
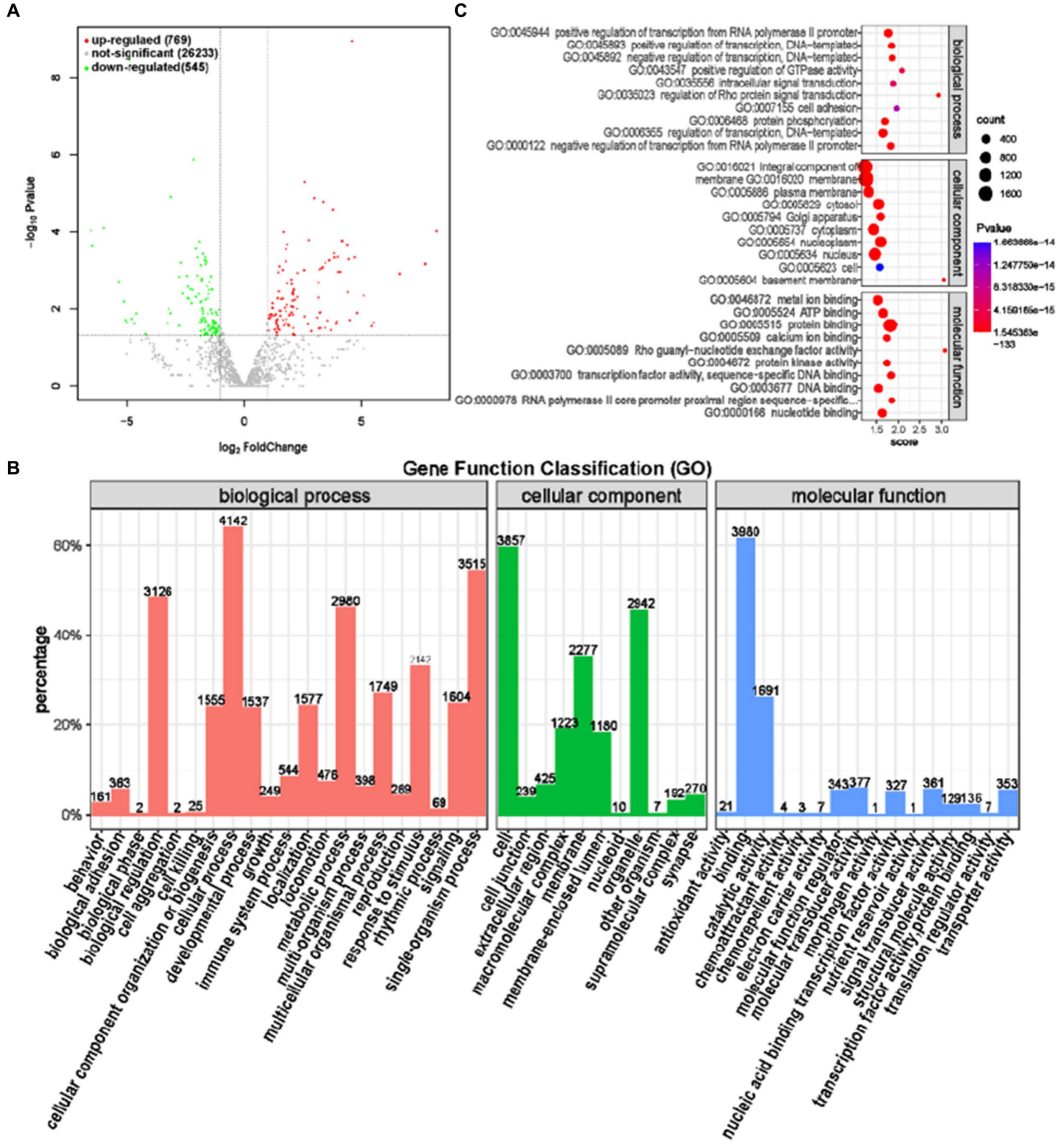
Differential expression analysis of miRNA between GAS group and CON group. **(A)** Volcano map of miRNA expression differences between GAS group and CON group. **(B)** GAS group and CON group differentially expressed miRNA GO annotation classification histogram. **(C)** The functional scatter plot shows a significant difference in miRNA enrichment between the GAS group and the CON group, with a top 10 * 3 degree (biological process, cellular component, and molecular function each with a top 10 degree).

MiRNA (MicroRNA) is a 17-24 nt single stranded non coding RNA molecule that can interact with mRNA to affect the stability and translation of target mRNA, regulate gene expression, cell growth, development, and other biological processes (26, 27). The study of miRNA sequencing is of great significance for understanding the mechanisms of biological growth and development, as well as the occurrence and development of diseases. A total of 1,314 differentially expressed miRNAs were screened between the GAS and CON groups, of which 769 were significantly upregulated and 545 were significantly downregulated (Figure 9A).

Functional annotation analysis of differentially expressed miRNA targeting mRNA genes revealed that genes differentially expressed in biological processes were mainly concentrated in cellular processes, single biological processes, biological regulation, and metabolic processes. The number of annotated genes in cell components with a high proportion was mainly concentrated in cells, organelles, and membranes, while protein binding and catalytic activity were higher in molecular functions annotated (Figure 9B). GO has three ontologies, with the highest enrichment level of top 10 in the molecular function, cellular component, and biological process of the gene (Table 6; Figure 9C). Compared with the CON group, the top 10 miRNA targeted mRNA genes in the GAS group were mainly enriched in transcriptional regulation, located in organelles and cell membranes, and the molecular functions were mainly protein, ATP, nucleotide, and DNA binding, consistent with the results of miRNA gene functional annotation.

**Table 6:** Gene functions of 30 significantly enriched differential miRNAs targeting mRNA in the GAS group.

GO.ID	Term	Ontology	GeneRatio^1^
GO:0006355	Regulation of transcription, DNA-templated	Biological process	416/6480
GO:0045944	Positive regulation of transcription from RNA polymerase II promoter	Biological process	328/6480
GO:0000122	Negative regulation of transcription from RNA polymerase II promoter	Biological process	233/6480
GO:0045893	Positive regulation of transcription, DNA-templated	Biological process	198/6480
GO:0006468	Protein phosphorylation	Biological process	235/6480
GO:0035023	Regulation of Rho protein signal transduction	Biological process	56/6480
GO:0045892	Negative regulation of transcription, DNA-templated	Biological process	160/6480
GO:0043547	Positive regulation of GTPase activity	Biological process	98/6480
GO:0035556	Intracellular signal transduction	Biological process	126/6480
GO:0007155	Cell adhesion	Biological process	111/6480
GO:0005654	Nucleoplasm	Cellular component	862/6480
GO:0005634	Nucleus	Cellular component	1115/6480
GO:0005829	Cytosol	Cellular component	808/6480
GO:0005737	Cytoplasm	Cellular component	893/6480
GO:0016020	Membrane	Cellular component	1696/6480
GO:0016021	Integral component of membrane	Cellular component	1522/6480
GO:0005886	Plasma membrane	Cellular component	759/6480
GO:0005794	Golgi apparatus	Cellular component	261/6480
GO:0005604	Basement membrane	Cellular component	46/6480
GO:0005623	Cell	Cellular component	226/6480
GO:0005515	Protein binding	Molecular function	1254/6480
GO:0005524	ATP binding	Molecular function	527/6480
GO:0046872	Metal ion binding	Molecular function	536/6480
GO:0000166	Nucleotide binding	Molecular function	408/6480
GO:0003700	Transcription factor activity, sequence-specific DNA binding	Molecular function	251/6480
GO:0003677	DNA binding	Molecular function	417/6480
GO:0005509	Calcium ion binding	Molecular function	240/6480
GO:0004672	Protein kinase activity	Molecular function	216/6480
GO:0005089	Rho guanyl-nucleotide exchange factor activity	Molecular function	53/6480
GO:0000978	RNA polymerase II core promoter proximal region sequence-specific DNA binding	Molecular function	168/6480

^1^GeneRatio: The number of target genes in this GO entry/the number of genes with GO annotations in the target gene. ^2^BgRatio: The number of genes in the GO entry of this entry/the number of genes with GO annotations in all genes.

## 4 Discussion

### 4.1 The effect of GAS on the growth performance and intestinal tissue morphology

Sheep (*Ovis aries*) alongside goat, are considered as poor man’s cow and they play an important role in the household economy of a poor farmer (28). Sheep provides wool along with good quality meat and milk due to which its role in the backyard and commercial farming cannot be neglected. Many approaches are being adapted for the proper growth and health of sheep among which use of natural products as therapeutic and growth promoting substances is gaining popularity from the past few decades (29–31). Research has found that GAS has strong functions such as antioxidation, detoxification, sterilization, disease prevention, and aiding digestion. Adding an appropriate amount of GAS to feed can not only improve feed taste and increase feed utilization, but also enhance animal immunity and prevent disease occurrence (5).

In pig breeding, adding garlic powder to the basic diet can improve growth performance, meat quality, and regulate gut microbiota (32). In the diet of calves, supplementing with garlic extract can significantly increase feed intake, feed conversion rate, and average daily weight gain (33). Previous studies have found that adding GAS to the diet of fattening sheep can improve the average daily weight gain and feed conversion efficiency of fattening sheep (19). This is consistent with the early experimental results of garlic and its extract allicin in livestock and aquatic animals (including poultry) (33, 34). The bioactive compounds present in GAS are essentially polyphenols, which have a positive impact on energy metabolism (35). Therefore, the effect of GAS on the growth performance of fattening sheep can be partially attributed to the improvement of energy utilization efficiency. On this basis, we further investigated the mechanism of adding GAS to the diet to improve the production performance of fattening sheep.

The tissue structure of the gastrointestinal tract plays an important regulatory role in the physiological, metabolic, and immune functions of animal bodies (36, 37). The results showed that adding 8% GAS to the diet had no significant effect on the gastrointestinal structure of fattening sheep (Supplementary Tables S7, S8; Supplementary Figures S1, S2). In addition, studies have found that GAS can protect gastrointestinal health and inhibit nematode infections (38). Meanwhile, GAS can increase the number of cellulose degrading bacteria in the rumen (39). This indicates that GAS can promote the growth and health of fattening sheep by protecting gastrointestinal health, improving gastrointestinal digestion and emission capacity, rather than regulating the growth performance of fattening sheep by regulating changes in gastrointestinal tissue structure.

### 4.2 The effect of GAS on the fecal microbiota

The analysis of microbial diversity at the fecal genus level between the GAS and CON groups showed significant changes in the abundance of four microbial communities. Compared with the CON group, the GAS group significantly upregulated Methanobrevibacter. Methanobrevibacter in the intestine can reduce the accumulation of intestinal gases, maintain an anaerobic environment in the hindgut, utilize nutrients in the intestinal cavity, and compete with other microorganisms for symbiosis. This is of great significance for stabilizing the intestinal microbiota and protecting intestinal health (38). The downregulated bacteria included *Akkermansia*, *Parasutterella*, and Guggenheimella. Akkermansia, as a bacterium that helps resist obesity, can be reduced within a reasonable range of abundance in the body (40). Reducing Parasutterella in the intestine is beneficial for maintaining gastrointestinal ecological stability and increasing gut microbiota diversity, reducing intestinal and metabolic diseases, and promoting intestinal health (41). This indicates that adding GAS to the diet is beneficial for the proliferation of probiotics in the gut of fattening sheep, reducing the number of harmful bacteria, thereby improving the composition of gut microbiota, promoting nutrient absorption, and promoting the growth performance and health of fattening sheep.

### 4.3 The effect of GAS on serum antioxidant indicators

The levels of superoxide dismutase, peroxidase, and catalase in serum are important indicators for measuring the body’s antioxidant capacity (42, 43). MDA is the final product of lipid peroxidation reaction, which can cause cross-linking and polymerization of life molecules such as proteins and nucleic acids, and has cytotoxicity (44, 45). The higher the content of malondialdehyde, the greater the harm to the body (46). Compared with the CON group, the GAS group significantly increased serum GSH-Px in fattening sheep, decreased MDA (*p* < 0.05), and slightly higher SOD and T-AOC than the CON group. This indicates that adding GAS to the diet can improve the antioxidant capacity of fattening sheep, reduce energy consumption and disease occurrence, and improve the growth efficiency of fattening sheep.

### 4.4 The effect of GAS on the metabolomics of serum and urine

Through serum and urine metabolomics analysis, it was found that metabolites such as taurine, taurocholate, glycine, and uric acid were significantly upregulated in the GAS group. Taurine, as a regulatory amino acid, has multiple functions such as antioxidant, regulating energy metabolism, detoxification, and promoting digestion and absorption (47). Taurocholate is formed by the combination of various substances such as taurine, glycine, and bile acids, promoting the digestion and absorption of fatty acids and vitamins (48). Uric acid is a natural antioxidant that can combat oxidative stress, eliminate oxygen free radicals, and maintain the body’s immune function (49). In addition, carbohydrates such as xylitol, mannose, and D-fructose are significantly reduced. Among them, an increase in the content of xylitol and mannose can stimulate the gastrointestinal tract to a certain extent, leading to intestinal ringing, loss of appetite, and diarrhea (50). High levels of fructose can cause an increase in ROS levels, leading to oxidative stress in the body (51).

The metabolic pathways that show significant changes in serum mainly include arginine and proline metabolism, taurine metabolism and taurine metabolism, and pyrimidine metabolism. The main metabolic pathways in urine are tryptophan metabolism, pyrimidine metabolism, and riboflavin metabolism. Arginine and proline metabolism participate in multiple metabolic pathways, synthesize immunoglobulins, and improve the body’s immune function (52). Taurine metabolism and secondary taurine metabolism enhance the digestion and absorption of lipids in the gastrointestinal tract, and exhibit anti-inflammatory and antioxidant effects in diseases that affect animal production, such as animal heat stress, gastrointestinal injury, and mastitis (53). Pyrimidine metabolism is of great significance in maintaining nucleic acid balance and repairing damaged DNA, providing nitrogen or carbon sources for microbial growth (54). Tryptophan metabolism enhances intestinal immune function and prevents intestinal diseases (55). Riboflavin metabolism is involved in the metabolism of proteins, fats, and carbohydrates in the body, promoting animal growth, improving animal production performance, and cold tolerance. It is an essential nutrient for animals (56).

Based on metabolomics analysis of serum and urine, adding GAS to the diet of fattening sheep can affect the levels of metabolites in serum and urine, increase beneficial metabolite levels, reduce harmful metabolites, and lead to changes in related metabolic pathways. These metabolites and metabolic pathways are related to energy metabolism, protein synthesis, disease treatment, and the immune capacity of fattening sheep, improving their antioxidant, antibacterial, and disease resistance abilities, thereby enhancing their growth performance.

### 4.5 The effect of GAS on transcriptomics of rumen epithelial tissue

CirRNA, as a novel type of RNA, has a circular structure and is involved in biological processes such as protein synthesis, immunity, and metabolism, playing an important role in organisms (57). LncRNA is a non coding RNA with a length greater than 200 nt, involved in the regulation of various life activities (58). MiRNA is a type of non-coding single stranded RNA molecule with a length of approximately 22 nt encoded by endogenous genes, involved in biological processes such as metabolism, immune response, and inflammation treatment (59). Through transcriptome sequencing of rumen epithelial tissue, the function and structure of genes were studied at the transcriptional level, revealing the effect of feeding GAS on gene expression in fattening sheep. Compared with the CON group, the GAS group showed significant upregulation of 7 cirRNAs and significant downregulation of 3 cirRNAs; 7 significantly upregulated lncRNAs and 39 significantly downregulated lncRNAs; 769 miRNAs were significantly upregulated, and 545 miRNAs were significantly upregulated. GO enrichment analysis was performed on differentially expressed cirRNAs, lncRNAs, and miRNAs, and it was found that these differentially expressed cirRNAs, lncRNAs, and miRNAs mainly involve functions such as energy metabolism, protein synthesis, antioxidant regulation, and immune response. This is consistent with the antioxidant, antibacterial, anti-inflammatory, and digestive promoting effects of GAS, confirming that GAS can promote the digestive ability of fattening sheep, improve their immune ability, and have important significance for the growth performance and health of fattening sheep.

In recent years, studies have found that adding a certain amount of garlic to the daily feed of various animals significantly improves feed conversion rate, daily feed intake, and survival rate of young offspring, effectively reducing the incidence of diseases (7, 8). GAS is a by-product of garlic production, and burning it as waste can easily pollute the environment. However, GAS is also rich in allicin and sulfides, which can resist the growth of various bacteria, viruses, and fungi, and have strong antibacterial effects (5). This experiment added GAS to the diet of fattening sheep as feed, and found that adding GAS can improve the growth performance of fattening sheep. We explained how GAS improves the growth performance of fattening sheep from the perspectives of serum antioxidant capacity, fecal microbiota analysis, serum and urine metabolites, and transcriptomics. This provides a reference for the application of garlic by-product GAS as feed in animal production, and also provides a theoretical basis for GAS to replace antibiotics and growth promoting agents in animal production.

## 5 Conclusion

In summary, adding 8% garlic peel to the diet improved the growth performance of fattening lamb and changed the composition of gut microbiota. Compared with the CON group, the GAS group significantly increased the relative abundance of Metanobrevibrater in the gut microbiota, while the relative abundance of Akkermansia, Parasutterella, and Guggenheimella was significantly reduced. Analysis of serum antioxidant indicators showed that compared with the CON group, the GAS group significantly increased serum GSH-Px and decreased MDA in the fattening lamb serum (*p* < 0.05), SOD and T-AOC slightly increased. Serum and urine metabolomics analysis showed that differential metabolites mainly included amino acids, fatty acids, dipeptides, and carbohydrates; the main signaling pathways included amino acid metabolism (taurine and taurine metabolism pathway, D-glutamine and D-glutamate metabolism pathway, arginine and proline metabolism pathway, cysteine and methionine metabolism pathway, tryptophan metabolism pathway, and biosynthesis of valine, leucine, and isoleucine), pyrimidine metabolism, and riboflavin metabolism pathway. These metabolic pathways were closely related to the improvement of growth performance and health status of fattening lamb. The transcriptome sequencing of rumen epithelial cells showed that the biological processes involved in circRNA, transcripts, and miRNA target genes mainly focused on cellular processes, biological regulation, and metabolic processes. The cellular locations were mainly cells and organelles, and molecular functions were mainly enriched in catalytic activity and protein connections. Functional enrichment analysis showed that differential expression was enriched in iron ion regulation, immune regulation, and transcriptional regulation functions. These results fully indicate that adding 8% GAS diet to fattening lamb can regulate protein synthesis and energy metabolism, improve gut microbiota composition, enhance serum antioxidant capacity, enhance body immunity, and improve growth performance and health level of fattening lamb. This has good reference value for healthy breeding.

## Data availability statement

The original contributions presented in the study are publicly available. These data can be found in the following repository: https://www.ncbi.nlm.nih.gov/bioproject, accession numbers: PRJNA1116814, PRJNA1050748, PRJNA1116343.

## Ethics statement

The animal study was approved by Animal Care Committee of Anhui Agriculture University (SYXK(Wan)2016-007). The study was conducted in accordance with the local legislation and institutional requirements.

## Author contributions

YX: Conceptualization, Data curation, Writing – original draft. MY: Data curation, Formal analysis, Investigation, Writing – review & editing. SS: Writing – review & editing. LW: Methodology, Project administration, Writing – review & editing. ZZ: Funding acquisition, Project administration, Writing – review & editing. YL: Software, Validation, Writing – review & editing. HC: Funding acquisition, Writing – review & editing.

## Footnotes

^1^ https://spssau.com/

## References

[1] Gul ST Alsayeqh AF. Probiotics as an alternative approach to antibiotics for safe poultry meat production. Pak Vet J. (2022) 42:285–91. doi: 10.29261/pakvetj/2022.061

[2] Caliskan GU Emin N. Protective efficacy of fresh and aged macerated garlic oils in safflower oil against intra-abdominal adhesions in rats. Pak Vet J. (2023) 43:290–6. doi: 10.29261/pakvetj/2023.030

[3] Del Rayo Camacho-Corona M Camacho-Morales A Góngora-Rivera F Escamilla-García E Morales-Landa JL Andrade-Medina M. Immunomodulatory effects of *Allium sativum* L. and its constituents against viral infections and metabolic diseases. Curr Top Med Chem. (2022) 22:109–31. doi: 10.2174/1568026621666211122163156, PMID: 34809549

[4] Jiang XY Liang JY Jiang SY Zhao P Tao F Li J. Garlic polysaccharides: a review on their extraction, isolation, structural characteristics, and bioactivities. Carbohydr Res. (2022) 518:108599. doi: 10.1016/j.carres.2022.108599, PMID: 35671643

[5] Singiri JR Swetha B Ben-Natan A Grafi G. What worth the garlic Peel. Int J Mol Sci. (2022) 23:2126. doi: 10.3390/ijms23042126, PMID: 35216242 PMC8875005

[6] Serrano-Jara D Rivera-Gomis J Tornel JA Jordán MJ Martínez-Conesa C Pablo MJC. Oregano essential oil and purple garlic powder effects on intestinal health, microbiota indicators and antimicrobial resistance as feed additives in weaning piglets. Animals (Basel). (2023) 14:111. doi: 10.3390/ani14010111 38200842 PMC10778277

[7] Aboubakr M Farag A Elfadadny A Alkafafy M Soliman A Elbadawy M. Allicin and lycopene possesses a protective effect against methotrexate-induced testicular toxicity in rats. Pak Vet J. (2023) 43:559–66. doi: 10.1007/s11356-023-28686-4

[8] Xu S Liao Y Wang Q Liu L Yang W. Current studies and potential future research directions on biological effects and related mechanisms of allicin. Crit Rev Food Sci Nutr. (2023) 63:7722–48. doi: 10.1080/10408398.2022.2049691, PMID: 35293826

[9] Huang W Yao C Liu Y Xu N Yin Z Xu W. Dietary Allicin improved the survival and growth of large yellow croaker (*Larimichthys crocea*) larvae via promoting intestinal development, alleviating inflammation and enhancing appetite. Front Physiol. (2020) 11:587674. doi: 10.3389/fphys.2020.587674, PMID: 33162901 PMC7583326

[10] Shi X Zhou X Chu X Wang J Xie B Ge J. Allicin improves metabolism in high-fat diet-induced obese mice by modulating the gut microbiota. Nutrients. (2019) 11:2909. doi: 10.3390/nu11122909 31810206 PMC6949904

[11] Nadeem MS Kazmi I Ullah I Muhammad K Anwar F. Allicin, an antioxidant and neuroprotective agent, ameliorates cognitive impairment. Antioxidants (Basel). (2021) 11:87. doi: 10.3390/antiox11010087 35052591 PMC8772758

[12] Bottje WG. Oxidative metabolism and efficiency: the delicate balancing act of mitochondria. Poult Sci. (2019) 98:4223–30. doi: 10.3382/ps/pey405, PMID: 30371897

[13] Jian Z Zeng L Xu T Sun S Yan S Yang L. Antibiotic resistance genes in bacteria: occurrence, spread, and control. J Basic Microbiol. (2021) 61:1049–70. doi: 10.1002/jobm.202100201 34651331

[14] Seidavi A Tavakoli M Asroosh F Scanes CG Abd el-Hack ME Naiel MAE. Antioxidant and antimicrobial activities of phytonutrients as antibiotic substitutes in poultry feed. Environ Sci Pollut Res Int. (2022) 29:5006–31. doi: 10.1007/s11356-021-17401-w, PMID: 34811612

[15] Yaqoob MU Wang G Wang M. An updated review on probiotics as an alternative of antibiotics in poultry – a review. Anim Biosci. (2022) 35:1109–20. doi: 10.5713/ab.21.0485, PMID: 35073660 PMC9262730

[16] Ding D Wang B Zhang X Zhang J Zhang H Liu X. The spread of antibiotic resistance to humans and potential protection strategies. Ecotoxicol Environ Saf. (2023) 254:114734. doi: 10.1016/j.ecoenv.2023.114734, PMID: 36950985

[17] Al-Khalaifa H Al-Nasser A Al-Surayee T Al-Kandari S Al-Enzi N Al-Sharrah T. Effect of dietary probiotics and prebiotics on the performance of broiler chickens. Poult Sci. (2019) 98:4465–79. doi: 10.3382/ps/pez282 31180128

[18] Tian M He X Feng Y Wang W Chen H Gong M. Pollution by antibiotics and antimicrobial resistance in LiveStock and poultry manure in China, and countermeasures. Antibiotics (Basel). (2021) 10:539. doi: 10.3390/antibiotics10050539 34066587 PMC8148549

[19] Zhu W Su Z Xu W Sun HX Gao JF Tu DF. Garlic skin induces shifts in the rumen microbiome and metabolome of fattening lambs. Animal. (2021) 15:100216. doi: 10.1016/j.animal.2021.100216, PMID: 34051409

[20] Kuziel GA Rakoff-Nahoum S. The gut microbiome. Curr Biol. (2022) 32:R257–r264. doi: 10.1016/j.cub.2022.02.023 35349808

[21] Zhou WY Cai ZR Liu J Wang DS Ju HQ Xu RH. Circular RNA: metabolism, functions and interactions with proteins. Mol Cancer. (2020) 19:172. doi: 10.1186/s12943-020-01286-3, PMID: 33317550 PMC7734744

[22] Panyod S Wu WK Ho CT Lu KH Liu CT Chu YL. Diet supplementation with Allicin protects against alcoholic fatty liver disease in mice by improving anti-inflammation and Antioxidative functions. J Agric Food Chem. (2016) 64:7104–13. doi: 10.1021/acs.jafc.6b02763, PMID: 27584700

[23] Bridges MC Daulagala AC Kourtidis A. LNCcation: lncRNA localization and function. J Cell Biol. (2021) 220:9045. doi: 10.1083/jcb.202009045, PMID: 33464299 PMC7816648

[24] Schmitz SU Grote P Herrmann BG. Mechanisms of long noncoding RNA function in development and disease. Cell Mol Life Sci. (2016) 73:2491–509. doi: 10.1007/s00018-016-2174-5, PMID: 27007508 PMC4894931

[25] Borlinghaus J Albrecht F Gruhlke M Nwachukwu I Slusarenko A. Allicin: chemistry and biological properties. Molecules. (2014) 19:12591–618. doi: 10.3390/molecules190812591, PMID: 25153873 PMC6271412

[26] Correia de Sousa M Gjorgjieva M Dolicka D Sobolewski C Foti M. Deciphering miRNAs' action through miRNA editing. Int J Mol Sci. (2019) 20:249. doi: 10.3390/ijms20246249, PMID: 31835747 PMC6941098

[27] Saliminejad K Khorram Khorshid HR Soleymani Fard S Ghaffari SH. An overview of microRNAs: biology, functions, therapeutics, and analysis methods. J Cell Physiol. (2019) 234:5451–65. doi: 10.1002/jcp.27486, PMID: 30471116

[28] Hussein SN Ibrahim AA Shukur MS. Molecular identification of Sarcocystis species in sheep (*Ovis aries*) and goats (*Capra hircus*) of Duhok Province, Iraq. Pakistan Vet J. (2023) 43:248–54. doi: 10.3390/ani12162048

[29] Okada S Inabu Y Miyamoto H Suzuki K Kato T Kurotani A. Estimation of silent phenotypes of calf antibiotic dysbiosis. Sci Rep. (2023) 13:6359. doi: 10.1038/s41598-023-33444-0, PMID: 37076584 PMC10115819

[30] Khan A Saleemi MK Ali F Abubakar M Hussain R Abbas RZ. Pathophysiology of peste des petits ruminants in sheep (Dorper & Kajli) and goats (Boer & Beetal). Microb Pathog. (2018) 117:139–47. doi: 10.1016/j.micpath.2018.02.009, PMID: 29427710

[31] Attia YA Hassan RA Addeo NF Bovera F Alhotan RA Al-qurashi AD. Effects of Spirulina platensis and/or *Allium sativum* on antioxidant status, immune response, gut morphology, and intestinal lactobacilli and coliforms of heat-stressed broiler chicken. Vet Sci. (2023) 10:678. doi: 10.3390/vetsci10120678 38133229 PMC10747519

[32] Janz JA Morel PCH Wilkinson BHP Purchas RW. Preliminary investigation of the effects of low-level dietary inclusion of fragrant essential oils and oleoresins on pig performance and pork quality. Meat Sci. (2007) 75:350–5. doi: 10.1016/j.meatsci.2006.06.027, PMID: 22063669

[33] Huang RH Qiu XS Shi FX Hughes CL Lu ZF Zhu WY. Effects of dietary allicin on health and growth performance of weanling piglets and reduction in attractiveness of faeces to flies. Animal. (2011) 5:304–11. doi: 10.1017/S1751731110001953, PMID: 22440775

[34] Gong HZ Wu M Lang WY Yang M Wang JH Wang YQ. Effects of laying breeder hens dietary β-carotene, curcumin, allicin, and sodium butyrate supplementation on the growth performance, immunity, and jejunum morphology of their offspring chicks. Poult Sci. (2020) 99:151–62. doi: 10.3382/ps/pez584, PMID: 32416796 PMC7587906

[35] Lan W Yang C. Ruminal methane production: associated microorganisms and the potential of applying hydrogen-utilizing bacteria for mitigation. Sci Total Environ. (2019) 654:1270–83. doi: 10.1016/j.scitotenv.2018.11.180 30841400

[36] Wang S Wang L Fan X Yu C Feng L Yi L. An insight into diversity and functionalities of gut microbiota in insects. Curr Microbiol. (2020) 77:1976–86. doi: 10.1007/s00284-020-02084-2 32535651

[37] Taylor AM Thompson SV Edwards CG Musaad SMA Khan NA Holscher HD. Associations among diet, the gastrointestinal microbiota, and negative emotional states in adults. Nutr Neurosci. (2020) 23:983–92. doi: 10.1080/1028415X.2019.1582578, PMID: 30794085

[38] Ding H Ao C Zhang X. Potential use of garlic products in ruminant feeding: a review. Anim Nutr. (2023) 14:343–55. doi: 10.1016/j.aninu.2023.04.011, PMID: 37635929 PMC10448032

[39] Zhong R Xiang H Cheng L Zhao C Wang F Zhao X. Effects of feeding garlic powder on growth performance, rumen fermentation, and the health status of lambs infected by gastrointestinal nematodes. Animals (Basel). (2019) 9:102. doi: 10.3390/ani9030102 30897693 PMC6466378

[40] Cani PD Depommier C Derrien M Everard A de Vos WM. *Akkermansia muciniphila*: paradigm for next-generation beneficial microorganisms. Nat Rev Gastroenterol Hepatol. (2022) 19:625–37. doi: 10.1038/s41575-022-00631-9, PMID: 35641786

[41] Wang Y Tao H Huang H Xiao Y Wu X Li M. The dietary supplement Rhodiola crenulata extract alleviates dextran sulfate sodium-induced colitis in mice through anti-inflammation, mediating gut barrier integrity and reshaping the gut microbiome. Food Funct. (2021) 12:3142–58. doi: 10.1039/D0FO03061A, PMID: 33729231

[42] Purba RAP Suong NTM Paengkoum S Schonewille JT Paengkoum P. Dietary inclusion of anthocyanin-rich black cane silage treated with ferrous sulfate heptahydrate reduces oxidative stress and promotes tender meat production in goats. Front Vet Sci. (2022) 9:969321. doi: 10.3389/fvets.2022.969321, PMID: 35990268 PMC9386371

[43] Ban C Paengkoum S Yang S Tian X Thongpea S Purba RAP. Feeding meat goats mangosteen (Garcinia mangostanaL.) peel rich in condensed tannins, flavonoids, and cinnamic acid improves growth performance and plasma antioxidant activity under tropical conditions. J Appl Anim Res. (2022) 50:307–15. doi: 10.1080/09712119.2022.2068557

[44] Purba RAP Paengkoum P. Exploring the phytochemical profiles and antioxidant, antidiabetic, and Antihemolytic properties of *Sauropus androgynus* dried leaf extracts for ruminant health and production. Molecules. (2022) 27:27. doi: 10.3390/molecules27238580 PMC9735450 36500671

[45] Purba RAP Paengkoum P. Farang (*Psidium guajava* L.) dried leaf extracts: phytochemical profiles, antioxidant, anti-diabetic, and anti-hemolytic properties for ruminant health and production. Molecules. (2022) 27:27. doi: 10.3390/molecules27248987 PMC9781826 36558117

[46] Del Rio D Stewart AJ Pellegrini N. A review of recent studies on malondialdehyde as toxic molecule and biological marker of oxidative stress. Nutr Metab Cardiovasc Dis. (2005) 15:316–28. doi: 10.1016/j.numecd.2005.05.003, PMID: 16054557

[47] Ma J Yang Z Jia S Yang R. A systematic review of preclinical studies on the taurine role during diabetic nephropathy: focused on anti-oxidative, anti-inflammation, and anti-apoptotic effects. Toxicol Mech Methods. (2022) 32:420–30. doi: 10.1080/15376516.2021.2021579, PMID: 34933643

[48] Yu X Meng X Yan Y Wang H Zhang L. Extraction of Naringin from pomelo and its therapeutic potentials against hyperlipidemia. Molecules. (2022) 27:27. doi: 10.3390/molecules27249033 PMC9783781 36558166

[49] Kumar B Kumawat BL Khan FA das GK Maurya SK Chandra P. Comparative analysis of biochemical, hormonal, and mineral compositions of preovulatory and cystic ovarian follicles in buffalo during the non-breeding season. Zygote. (2023) 31:246–52. doi: 10.1017/S0967199423000084, PMID: 36919850

[50] Zhang Y Vera JM Xie D Serate J Pohlmann E Russell JD. Multiomic fermentation using chemically defined synthetic Hydrolyzates revealed multiple effects of lignocellulose-derived inhibitors on cell physiology and xylose utilization in *Zymomonas mobilis*. Front Microbiol. (2019) 10:2596. doi: 10.3389/fmicb.2019.02596, PMID: 31787963 PMC6853872

[51] Loh CH Kuo W-W Lin S-Z Shih C-Y Lin P-Y Situmorang JH. Corrigendum to “PKC-δ-dependent mitochondrial ROS attenuation is involved as 9-OAHSA combats lipoapotosis in rat hepatocytes induced by palmitic acid and in Syrian hamsters induced by high-fat high-cholesterol high-fructose diet” [Toxicology and Applied Pharmacology, 470, (2023), 116557]. Toxicol Appl Pharmacol. (2023) 476:116658. doi: 10.1016/j.taap.2023.116658 37207915

[52] Wu G Bazer FW Burghardt RC Johnson GA Kim SW Knabe DA. Proline and hydroxyproline metabolism: implications for animal and human nutrition. Amino Acids. (2011) 40:1053–63. doi: 10.1007/s00726-010-0715-z, PMID: 20697752 PMC3773366

[53] Wang Z Ohata Y Watanabe Y Yuan Y Yoshii Y Kondo Y. Taurine improves lipid metabolism and increases resistance to oxidative stress. J Nutr Sci Vitaminol (Tokyo). (2020) 66:347–56. doi: 10.3177/jnsv.66.347, PMID: 32863308

[54] Garavito MF Narváez-Ortiz HY Zimmermann BH. Pyrimidine metabolism: dynamic and versatile pathways in pathogens and cellular development. J Genet Genomics. (2015) 42:195–205. doi: 10.1016/j.jgg.2015.04.004, PMID: 26059768

[55] Xue C Li G Zheng Q Gu X Shi Q Su Y. Tryptophan metabolism in health and disease. Cell Metab. (2023) 35:1304–26. doi: 10.1016/j.cmet.2023.06.004, PMID: 37352864

[56] Suwannasom N Kao I Pruß A Georgieva R Bäumler H. Riboflavin: the health benefits of a forgotten natural vitamin. Int J Mol Sci. (2020) 21:950. doi: 10.3390/ijms21030950, PMID: 32023913 PMC7037471

[57] Liu CX Chen LL. Circular RNAs: characterization, cellular roles, and applications. Cell. (2022) 185:2390. doi: 10.1016/j.cell.2022.06.001, PMID: 35750036

[58] Gudenas BL Wang L. Prediction of LncRNA subcellular localization with deep learning from sequence features. Sci Rep. (2018) 8:16385. doi: 10.1038/s41598-018-34708-w, PMID: 30401954 PMC6219567

[59] Mehta A Baltimore D. MicroRNAs as regulatory elements in immune system logic. Nat Rev Immunol. (2016) 16:279–94. doi: 10.1038/nri.2016.40, PMID: 27121651

