# Supplementary material for: The regulatory mechanism of garlic skin improving the growth performance of fattening sheep through metabolism and immunity
Source: Front Vet Sci. 2024 May 30;11:1409518. doi: 10.3389/fvets.2024.1409518 (PMC11171129; doi:10.3389/fvets.2024.1409518)
Supplement: Supplementary file 1 [file Data_Sheet_1.PDF]

Supplementary Material

The regulatory mechanism of garlic skin improving the growth performance of fattening lamb through metabolism and immunity

Yongjie Xu[1†], Mingliang Yi[1†], Shixin Sun[1], Lei Wang[1], Zijun Zhang[1,2], Yinghui Ling[1,2], Hongguo Cao [1,2]*

*Corresponding Author: Hongguo Cao

E-mail: caohongguo1@ahau.edu.cn

[1]College of Animal Science and Technology, Anhui Agricultural University, Hefei 230036, P.R. China

[2] Anhui Province Key Laboratory of Local Livestock and Poultry Genetic Resource Conservation and Bio-breeding, Anhui Agricultural University, Hefei, 230036, P.R. China

[a] These authors contributed equally to this work.

Supplementary Tables and Figures

Supplementary Table S1. Significant differences in metabolites between GAS group and CON group in serum of fattening sheep.

Name	MZ[1]	R.T[2](min)	VIP[3]	P-value[4]	FC[5]
**Negative**					
Acetohydroxamic acid	74.025	172.453	1.224	0.047	0.595
ketoisocaproic acid	129.056	46.747	1.514	0.027	1.502
4-Nitrophenol	138.019	38.009	1.525	0.000	0.520
L-Glutamate	146.045	293.426	1.318	0.030	1.248
DL-3-Phenyllactic acid	147.044	106.107	1.458	0.026	0.680
Formylanthranilic acid	164.035	68.879	1.689	0.004	0.503
resorcinol	169.051	34.829	1.760	0.001	2.972
Glycerol 3-phosphate	171.006	372.989	1.730	0.001	0.552
L-Citrulline	174.088	401.489	1.422	0.012	0.857
Urocanic acid	174.995	24.518	1.425	0.030	1.825
L-Ascorbic acid	175.024	294.968	1.543	0.038	2.027
Allantoate/Allantoic acid	175.047	333.298	2.055	0.000	0.286
Beta-Alanine	177.091	48.385	1.930	0.002	0.300
D-Fructose	179.055	379.602	1.335	0.040	0.822
Dihydrothymine	187.072	372.274	2.037	0.000	0.252
Salicyluric acid	194.045	156.787	1.309	0.047	1.355
Citramalic acid	207.050	287.748	1.737	0.002	1.895
Pyrocatechol	219.065	34.008	1.284	0.050	1.294
Lipoamide	221.081	47.500	1.732	0.017	0.171
N-(omega)-Hydroxyarginine	227.056	266.059	1.683	0.001	1.865
Confertifoline	233.155	30.742	1.042	0.038	2.105
Glucosamine	238.092	296.552	1.747	0.002	0.577
Cytidine	242.078	231.235	1.250	0.047	0.816

Phosphorylcholine	242.079	373.433	2.025	0.000	0.549
Stavudine	245.056	167.719	1.324	0.028	0.559
Muramic acid	250.093	352.874	1.747	0.004	0.397
5'-O-methylthymidine	256.104	144.418	1.839	0.008	0.112
Alpha-N-Phenylacetyl-L-glutamine	263.103	204.124	1.240	0.044	0.669
(R)-mevalonic acid 5-Phosphate	265.089	26.224	1.943	0.006	4.162
2'-O-methylinosine	281.088	143.847	1.531	0.019	1.240
Salidroside	281.098	252.552	1.331	0.046	1.321
D-Sorbitol 6-phosphate	283.126	28.554	1.399	0.011	1.391
N4-Acetylcytidine	284.088	158.482	1.692	0.020	3.397
N-Acetylneuraminic acid	290.087	325.584	1.446	0.022	0.857
2'-O-Methyluridine	295.033	169.445	1.220	0.041	0.794
3'-O-methylguanosine	296.099	189.024	1.810	0.001	1.250
Eicosapentaenoic Acid	301.216	43.507	1.265	0.042	0.765
Dioxybenzone	303.089	32.685	1.733	0.004	2.117
Arachidonic Acid (peroxide free)	303.233	43.085	1.390	0.018	0.720
3-Deoxy-2-keto-6-phosphogluconic acid	317.142	32.798	1.410	0.018	0.626
Norethindrone Acetate	339.198	60.102	1.420	0.030	0.795
Behenic acid	339.326	40.951	1.589	0.004	0.763
Sucrose	342.118	315.467	2.005	0.000	0.219
Maltitol	344.133	260.680	1.377	0.019	0.631
Tetracosanoic acid	367.356	40.381	1.528	0.003	0.704
Hexacosanoic acid	395.387	39.835	1.302	0.014	0.633
Sunitinib	397.204	290.737	1.207	0.038	1.5388
Cortisone acetate	401.198	41.957	1.264	0.039	1.269
Sedoheptulose	419.132	207.390	1.507	0.041	1.856
N-Formylmethionyl-Leucylphenylalanine	436.197	294.122	1.772	0.000	0.278
Glycochenodeoxycholate	448.304	189.572	1.653	0.026	2.305
MK 571	573.126	333.185	1.923	0.002	0.145
1-Palmitoyl-2-oleoyl-sn-glycero-3-phosphate	673.477	124.828	1.290	0.040	0.856
PS(16:0/16:0)	734.515	49.684	1.656	0.001	0.423
**Postive**					
Urea	61.040	105.905	1.361	0.015	0.850
Pyrrolidine	72.081	306.027	1.451	0.004	1.470
Acetohydroxamic acid	76.039	339.402	1.378	0.032	0.679
Glycine	76.039	368.020	1.818	0.000	0.427
Diethanolamine	88.076	211.625	1.994	0.001	19.516
L-Alanine	90.055	334.322	1.342	0.020	0.888
Cytosine	112.051	231.207	1.352	0.032	0.793
N-Methylhydantoin	115.049	334.322	1.383	0.012	0.881

Acetylglycine	118.049	368.037	1.875	0.000	0.292
Taurine	126.021	281.184	1.232	0031	1.236
Creatine	132.077	334.322	1.449	0.012	0.870
Adenine	136.061	154.021	1.871	0.010	0.395
Larixinic Acid	144.064	252.795	1.914	0.000	0.234
Pyruvaldehyde	145.049	293.416	1.587	0.103	2.241
Dihydro-4,4-dimethyl-2,3-furandione	146.080	321.216	1.616	0.005	0.818
Levonordefrin	148.074	171.378	1.366	0.016	0.716
DL-a-Hydroxybutyric acid	149.022	33.168	2.009	0.000	0.438
trans-cinnamate	149.058	245.101	1.332	0.033	1.138
2-Ethoxyethanol	151.095	63.928	1.689	0.002	0.530
N1-Methyl-2-pyridone-5-carboxamide	153.064	85.952	1.243	0.040	0.748
Scopoline	156.101	351.744	1.345	0.035	0.737
Caproic acid	158.117	47.430	1.870	0.001	2.529
D-Glucuronate	159.027	208.993	1.503	0.005	0.589
Acetyl-DL-Valine	160.096	313.750	1.446	0.001	0.432
1-Aminocyclopropanecarboxylic acid	162.075	252.795	1.955	0.000	0.354
beta-Hydroxybutyrate	168.065	252.499	1.982	0.000	0.261
L-Pipecolic acid	171.112	252.565	1.767	0.008	0.250
.beta.-Cyano-L-alanine	175.070	209.013	1.631	0.003	0.648
L-Arginine	175.119	516.673	1.319	0.026	0.709
3,4-Dihydroxyphenylacetic acid	186.075	252.614	1.992	0.000	0.308
Valproic acid	186.147	37.645	1.718	0.011	1.924
Kynuramine	187.089	283.929	2.008	0.000	0.116
Kynurenic acid	190.048	173.800	1.436	0.022	0.691
Phenylacetylglycine	194.080	172.174	1.187	0.028	0.601
Leu-Ala	203.138	330.389	1.614	0.004	0.729
Pyridostigmine cation	204.086	252.598	1.981	0.000	0.277
Acetylcarnitine	204.123	293.416	1.652	0.026	2.795
Pelletierine	205.126	293.423	1.661	0.028	3.006
Pyridoxine	211.106	85.616	1.688	0.001	0.568
DL-Vanillylmandelic acid	216.085	352.428	1.607	0.007	0.542
Thr-Val	218.123	390.979	1.421	0.025	0.549
Simazine	219.115	99.908	1.366	0.048	1.789
D-Glucono-1,5-lactone	220.080	394.621	1.538	0.002	0.409
Dulcitol	224.112	283.397	1.811	0.001	0.424
N-(omega)-Hydroxyarginine	232.139	426.585	1.659	0.010	0.642
D-gluconate	238.091	368.003	1.909	0.000	0.239
Gly-Glu	246.107	365.740	1.661	0.001	0.556
Val-Gln	246.144	313.231	1.921	0.000	0.137
Propazine	247.144	187.287	1.287	0.021	2.213

Miglitol	249.148	187.303	1.407	0.029	1.850
His-Thr	257.123	346.138	1.307	0.032	0.669
5-Methylcytidine	258.107	194.028	1.192	0.040	0.839
Glycerophosphocholine	258.110	372.063	1.734	0.000	0.437
Ribothymidine	259.091	328.566	1.262	0.039	0.817
2-Methyl-3-hydroxybutyric acid	259.119	28.241	1.542	0.006	1.616
1,7-Dimethyluric acid	260.073	370.201	1.884	0.000	0.309
Flumequine	261.076	370.429	1.660	0.001	0.559
Palmitic acid	274.273	53.952	1.660	0.033	0.273
cis-9-Palmitoleic acid	277.215	46.768	1.421	0.019	1.424
Phthalic acid Mono-2-ethylhexyl Ester	279.158	33.252	1.994	0.000	0.385
Val-Tyr	280.138	313.221	1.723	0.000	0.344
Ile-Ser	282.142	313.196	1.859	0.000	0.485
Guanosine	284.098	251.740	1.320	0.029	0.592
Ile-Asn	284.105	371.607	1.648	0.001	0.532
N4-Acetylcytidine	286.102	158.142	1.596	0.028	2.837
Ile-Arg	288.202	323.000	1.588	0.003	0.643
Arg-Ile	288.201	348.125	1.318	0.020	0.677
Gamma-Glutamylcysteine	292.101	409.542	1.782	0.001	0.556
Ile-Tyr	294.153	340.460	1.532	0.017	0.493
Gly-Arg	295.149	433.332	1.746	0.001	0.430
Ile-Thr	296.158	294.156	1.885	0.000	0.421
Sphingosine	300.288	111.232	1.325	0.036	0.659
4-Oxoretinol	301.215	35.353	1.801	0.000	1.831
Tyr-Lys	309.164	439.953	1.915	0.000	0.374
Omeprazole	310.094	364.899	1.829	0.000	0.365
Pro-Met	310.112	409.319	1.812	0.000	0.348
Phe-Cys	310.127	280.996	1.988	0.000	0.336
Tyr-Phe	311.132	370.987	1.999	0.000	0.350
Met-Tyr	312.110	306.241	1.865	0.000	0.307
Argininosuccinic acid	313.113	306.241	1.889	0.000	0.385
5-Hydroxymethylcytidine	315.133	36.854	1.526	0.034	4.484
His-Tyr	318.128	410.891	1.830	0.000	0.483
Linoleoyl ethanolamide	324.288	36.798	1.226	0.044	1.160
Zolmitriptan	326.125	434.955	1.759	0.001	0.440
N-Oleoylethanolamine	326.304	35.303	1.329	0.040	1.431
D-Ribulose 1,5-bisphosphate	328.017	352.737	1.585	0.012	0.559
Phe-Tyr	328.138	280.875	1.963	0.000	0.250
(-)-Medicarpin	331.110	372.176	1.939	0.000	0.267
beta-Octylglucoside	337.159	354.300	1.810	0.000	0.344
Arg-Tyr	337.172	450.647	1.869	0.000	0.276
Arg-Cys	338.154	393.075	1.992	0.000	0.317
Enoxacin	338.162	353.817	1.816	0.000	0.377

Arg-Thr	339.175	451.272	1.908	0.000	0.358
Famciclovir	344.133	315.326	1.957	0.000	0.204
Visnadin	353.132	293.863	1.969	0.000	0.286
(+-)5,6-DHET	356.277	83.476	1.845	0.010	0.179
Behenic acid	358.366	51.570	1.501	0.009	0.453
20-Hydroxyarachidonic acid	362.268	43.412	1.564	0.024	0.573
Phenoxybenzamine	367.148	282.726	1.908	0.000	0.267
Lathosterol	369.349	34.268	1.334	0.031	0.648
Tyr-Glu	371.143	294.147	1.951	0.003	0.211
Cortexolone	385.170	364.673	1.789	0.002	0.351
Glycochenodeoxycholate	432.308	189.862	1.582	0.019	2.051
Verapamil	447.308	46.614	1.798	0.006	7.440
Glycodeoxycholic acid	450.319	189.964	1.417	0.031	2.119
Troglitazone	459.206	338.329	1.911	0.014	0.114
Taurocholate	480.276	179.565	1.445	0.048	2.958
1-Stearoyl-2-arachidonoyl-sn-glycerol	627.532	46.640	1.998	0.000	0.111
Sphingomyelin (d18:1/18:0)	794.602	130.607	1.341	0.032	0.647
PC(20:5(5Z,8Z,11Z,14Z,17Z)/20:5(5Z,8Z, 11Z,14Z,17Z))	809.542	46.498	1.117	0.035	40.528
N-Docosanoyl-4-sphingenyl-1-O-phosphorylcholine	809.648	153.315	1.318	0.025	0.760
N-Tetracosanoyl-4-sphingenyl-1-O-phosphorylcholine	814.683	154.032	1.218	0.049	0.684

[1]MZ = mass-to-charge ratio.

[2]R.T = represents retention time.

[3]VIP >1 and [4]P-value<0.05 are listed in the table. P -values were calculated according to Student's T-test (n=6).

[5]FC = fold change. If the fold change value is less than 1, it means that there is less metabolite in the GAS group than in the CON group.

Supplementary Table S2. Pathway analysis of serum metabolomics in GAS and CON groups of fattening sheep.

Pathway	Total	Hits[1]	Raw P[2]	Impact[3]	Hits compounds
Arginine and proline metabolism	44	5	0.006	0.121	Argininosuccinic acid cpd:C03406; L-Arginine cpd:C00062; N-(o)-Hydroxyarginine cpd:C05933; Creatine cpd:C00300; Urea cpd:C00086
Taurine and hypotaurine metabolism	7	2	0.015	0.750	Taurine cpd:C00245; Taurocholic acid cpd:C05122
Primary bile acid biosynthesis	46	4	0.037	0.119	Glycine cpd:C00037; Taurine cpd:C00245; Chenodeoxycholic acid glycine conjugate cpd:C05466; Taurocholic acid cpd:C05122
Glycine, serine and threonine metabolism	32	3	0.058	0.292	Glycine cpd:C00037; Creatine cpd:C00300; Pyruvaldehyde cpd:C00546
Cyanoamino acid metabolism	6	1	0.157	0	Glycine cpd:C00037
Methane metabolism	9	1	0.226	0	Glycine cpd:C00037
Nitrogen metabolism	9	1	0.226	0	Glycine cpd:C00037
Vitamin B6 metabolism	9	1	0.226	0.078	Pyridoxine cpd:C00314
Ascorbate and aldarate metabolism	9	1	0.226	0.400	D-Glucuronic acid cpd:C00191
Purine metabolism	68	2	0.297	0.009	Guanosine cpd:C00387; Adenine cpd:C00147; Urea cpd:C00086
Nicotinate and nicotinamide metabolism	13	1	0.310	0	N1-Methyl-2-pyridone-5-carboxamide cpd:C05842
Biosynthesis of unsaturated fatty acids	42	1	0.331	0	Behenic acid cpd:C08281; Palmitic acid cpd:C00249
Pentose and glucuronate interconversions	15	1	0.349	0	D-Glucuronic acid cpd:C00191
Pentose phosphate pathway	19	1	0.420	0	Gluconolactone cpd:C00198
Propanoate metabolism	20	1	0.436	0	2-Hydroxybutyric acid cpd:C05984
Sphingolipid metabolism	21	1	0.452	0.053	Sphingosine cpd:C00319
Pyruvate metabolism	22	1	0.468	0.054	Pyruvaldehyde cpd:C00546

Starch and sucrose metabolism	23	1	0.483	0	D-Glucuronic acid cpd:C00191
Alanine, aspartate and glutamate metabolism	23	1	0.483	0.020	Argininosuccinic acid cpd:C03406
Porphyrin and chlorophyll metabolism	25	1	0.512	0	Glycine cpd:C00037
Galactose metabolism	26	1	0.526	0	Galactitol cpd:C01697
Glutathione metabolism	26	1	0.526	0.006	Glycine cpd:C00037
Fatty acid elongation in mitochondria	27	1	0.539	0	Palmitic acid cpd:C00249
Aminoacyl-tRNA biosynthesis	64	2	0.544	0	L-Arginine cpd:C00062; Glycine cpd:C00037
Inositol phosphate metabolism	28	1	0.553	0	D-Glucuronic acid cpd:C00191
Glycerophospholipid metabolism	29	1	0.565	0.024	Glycerophosphocholine cpd:C00670
Steroid biosynthesis	35	1	0.635	0.064	Lathosterol cpd:C01189
Fatty acid biosynthesis	38	1	0.666	0	Palmitic acid cpd:C00249
Fatty acid metabolism	39	1	0.675	0	Palmitic acid cpd:C00249
Tyrosine metabolism	42	1	0.703	0.001	3,4-Dihydroxybenzeneacetic acid cpd:C01161
Drug metabolism - cytochrome P450	56	1	0.803	0	Valproic acid cpd:C07185
Pyrimidine metabolism	37	3	0.027	0.013	Cytidine cpd:C00475; Dihydrothymine cpd:C00906; Beta-Alanine cpd:C00099
D-Glutamine and D-glutamate metabolism	5	1	0.087	1	L-Glutamic acid cpd:C00025
Histidine metabolism	14	1	0.225	0.130	Urocanic acid cpd:C00785
Pantothenate and CoA biosynthesis	15	1	0.239	0	Beta-Alanine cpd:C00099
Terpenoid backbone biosynthesis	15	1	0.239	0.172	Mevalonic acid-5P cpd:C01107
beta-Alanine metabolism	17	1	0.267	0.444	Beta-Alanine cpd:C00099
Glycerolipid metabolism	18	1	0.280	0.026	Glycerol 3-phosphate cpd:C00093
Butanoate metabolism	20	1	0.306	0	L-Glutamic acid cpd:C00025
Arachidonic acid metabolism	36	1	0.484	0.326	Arachidonic acid cpd:C00219
Amino sugar and nucleotide sugar	37	1	0.493	0	Glucosamine cpd:C00329

metabolism					
Tryptophan metabolism	41	1	0.530	0.010	Formylanthranilic acid cpd:C05653

[1] Hits represent the number of significantly different ruminal metabolites matched in one pathway.

[2] P is the original P value obtained by pathway analysis.

[3] Impact is the influencing factor of the pathway obtained by topology analysis.

Supplementary Table S3. Significant differences in metabolites between GAS and CON groups in urine of fattening sheep.

Name	MZ	R.T(min)	VIP	Pvalue	FC
**Negative**					
Hydroxyacetone	133.048	41.372	1.590	0.044	0.652
4-Aminosalicylic acid	134.024	32.901	1.810	0.047	0.634
3-Aminopropanesulphonic Acid	138.022	246.723	2.324	0.007	13.663
Ethosuximide	140.071	228.762	1.785	0.036	0.566
3-Isopropylmalate	157.049	131.265	1.765	0.022	0.546
4-Hydroxycoumarin	161.023	48.481	2.015	0.005	0.510
Citric acid	191.024	241.599	2.185	0.018	10.170
Shikimate	195.029	281.599	1.642	0.040	0.700
6-Hydroxynicotinic acid	198.039	50.678	1.712	0.042	0.680
Caprylic acid	203.127	205.179	1.499	0.041	0.567
L-Serine	209.080	192.562	1.760	0.015	0.517
2-keto-D-Gluconic acid	210.059	72.716	1.738	0.030	0.563
Oxypurinol	211.046	37.478	1.948	0.003	0.466
2'-Deoxyuridine	227.069	127.288	1.704	0.013	0.538
gamma-Glutamyl-L-methionine	259.071	269.993	1.823	0.016	0.458
DL-Homocystine	284.078	420.722	1.917	0.011	0.627
L-Cystine	299.034	246.579	2.030	0.014	7.999
Gemfibrozil	309.167	103.414	1.732	0.003	0.546
Maltitol	325.111	393.745	1.534	0.040	0.626
Clozapine	325.128	46.372	1.631	0..022	0.631
Formononetin	327.083	104.059	1.968	0.015	0.585
Phloretin	333.097	180.420	1.781	0.044	0.573
Adenylsuccinic acid	462.058	36.029	1.486	0.040	0.717
Topiramate	677.175	271.569	2.024	0.035	0.196
Flavin mononucleotide (FMN)	455.095	47.191	1.965	0.012	0.548
2-Deoxyribose 5-phosphate	273.041	87.722	1.910	0.016	0.409
Uridine	281.017	296.587	2.065	0.019	0.558
Postive					
3,3-Dimethylacrylic acid	101.058	273.386	1.726	0.046	0.687
gamma-Aminobutryic acid	121.099	34.181	2.070	0.005	0.678
Urocanic acid	139.049	267.551	1.663	0.031	1.511
Xylitol	153.072	283.425	2.108	0.003	0.713
3-Methoxytyramine	168.106	117.170	1.521	0.031	0.594
Indoleacrylic acid	170.059	168.350	1.856	0.036	0.444
L-Kynurenine	173.069	209.563	1.538	0.048	0.712
Succinate	179.051	33.044	1.824	0.013	0.480
3-Methoxybenzoic acid	191.015	107.439	2.314	0.007	3.125
4-Nitroquinoline-1-oxide	191.043	101.540	1.706	0.014	1.483
5-Hydroxyindoleacetate	192.065	35.225	1.813	0.021	0.498

Phenylacetylglycine	194.077	251.791	1.345	0.049	0.691
3-Guanidinopropanoate	198.021	40.265	1.719	0.026	0.535
.beta.-Citronellol	201.122	321.239	2.584	0.000	0.614
Gly-Gln	203.089	245.191	1.739	0.029	0.577
Dimethylbenzimidazole	213.035	90.616	1.766	0.045	0.387
2-Isopropylmalic acid	218.101	322.592	1.590	0.045	0.684
Acetyl-DL-Leucine	234.132	265.888	1.845	0.028	0.569
L-Valine	235.167	49.399	1.739	0.012	0.564
D-Erythrose 4-phosphate	242.043	234.928	2.402	0.010	9.057
Prometon	248.147	393.769	2.029	0.004	6.397
1,2-Diacetylhydrazine	250.152	393.316	1.713	0.015	1.850
1,2,3-Benzenetriol	253.068	401.409	1.794	0.010	0.738
Ile-Thr	255.133	485.522	1.892	0.038	0.433
Glu-Lys	258.143	392.461	1.582	0.033	0.668
Ile-Tyr	259.142	194.579	1.863	0.050	0.301
Gly-Glu	265.106	228.713	1.742	0.021	0.725
5'-Deoxyadenosine	269.135	234.703	2.297	0.001	0.691
Gly-Arg	276.106	286.154	1.652	0.020	0.679
Nortriptyline	286.163	181.948	1.407	0.048	0.554
Ser-Lys	310.052	338.549	1.852	0.010	0.407
Lys-Trp	315.181	316.354	1.638	0.048	0.670
Nateglinide	318.199	276.860	1.926	0.028	0.762
2-Oxoadipic acid	321.086	46.427	1.771	0.033	0.602
Photinus luciferin	322.036	211.080	2.370	0.011	35.554
5-Aminosalicylic Acid	324.114	385.726	2.053	0.005	0.601
Eicosapentaenoic acid	363.249	49.309	1.735	0.040	0.642
Isopentenyladenosine	374.127	385.388	2.094	0.010	0.616
Aminopterin	458.180	271.390	2.031	0.011	0.642
Mimosine	521.200	62.494	1.930	0.021	0.506
Vitexin	433.117	197.085	1.679	0.037	0.620

Supplementary Table S4. Pathway analysis of urine metabolomics in GAS and CON groups of fattening sheep.

Pathway	Total	Hits	Raw P	Impact	Hits compounds
Tryptophan metabolism	41	3	0.010	0.051	5-Hydroxyindoleacetic acid cpd:C05635; Oxoadipic acid cpd:C00322; L-Kynurenine cpd:C00328
Valine, leucine and isoleucine biosynthesis	11	1	0.120	0.333	L-Valine cpd:C00183
Histidine metabolism	14	1	0.150	0.130	Urocanic acid cpd:C00785
Pentose and glucuronate interconversions	15	1	0.160	0	D-Xylitol cpd:C00379
Pantothenate and CoA biosynthesis	15	1	0.160	0	L-Valine cpd:C00183
Pentose phosphate pathway	19	1	0.198	0	D-Erythrose 4-phosphate cpd:C00279
Propanoate metabolism	20	1	0.208	0	Succinic acid cpd:C00042
Butanoate metabolism	20	1	0.208	0	Succinic acid cpd:C00042
Citrate cycle (TCA cycle)	20	1	0.208	0.026	Succinic acid cpd:C00042
Lysine degradation	20	1	0.208	0.090	Oxoadipic acid cpd:C00322
Alanine, aspartate and glutamate metabolism	23	1	0.235	0	Succinic acid cpd:C00042
Valine, leucine and isoleucine degradation	38	1	0.360	0	L-Valine cpd:C00183
Biosynthesis of unsaturated fatty acids	42	1	0.389	0	Eicosapentaenoic acid cpd:C06428
Tyrosine metabolism	42	1	0.389	0.031	3-Methoxytyramine cpd:C05587
Aminoacyl-tRNA biosynthesis	64	1	0.531	0	L-Valine cpd:C00183
Cysteine and methionine metabolism	28	2	0.039	0.023	L-Serine cpd:C00065; L-Cystine cpd:C00491
Pyrimidine metabolism	37	2	0.065	0.079	Uridine cpd:C00299; Deoxyuridine cpd:C00526
Cyanoamino acid metabolism	6	1	0.067	0	L-Serine cpd:C00065

Methane metabolism	9	1	0.099	0.400	L-Serine cpd:C00065
Riboflavin metabolism	11	1	0.120	0.333	Flavin Mononucleotide cpd:C00061
Sphingolipid metabolism	21	1	0.217	0	L-Serine cpd:C00065
Glycine, serine and threonine metabolism	32	1	0.312	0.243	L-Serine cpd:C00065
Purine metabolism	68	1	0.554	0	Adenylsuccinic acid cpd:C03794

Supplementary Table S5. Analysis of significant differential expression of circRNA between GAS group and CON group.

**circRNA id**[1]	**MeanRPKM**[2] **(GAS)**	**MeanRPKM (CON)**	**log2FoldChange**[3]	**pValue**[4]
**Up**				
circRNA01756	303.543	0.000	21.533	0.048
circRNA01459	305.796	0.000	21.544	0.040
circRNA02866	156573.368	0.000	30.544	0.005
circRNA00924	676.224	0.000	22.689	0.022
circRNA01508	1619.264	0.000	23.949	0.046
circRNA01358	88.199	0.000	19.750	0.037
circRNA01332	957.052	0.000	23.190	0.027
**Down**				
circRNA01671	1275.178	4627.542	-1.860	0.027
circRNA04388	0.000	285.001	-21.443	0.046
circRNA03893	6.821	25.231	-1.887	0.048

[1] circRNA ID: Transcript number.

[2] MeanTPM: Expression level of grouping.

[3] log2FoldChange: log2 value of difference multiple.

[4] Pvalue: Statistical significance test indicators.

Supplementary Table S6. Significant differential expression analysis of transcripts (lncRNA, mRNA) between GAS group and CON group.

**Transcript id**[1]	**MeanTPM (GAS)**	**MeanTPM (CON)**	**log2FoldChange**	**Pvalue**
**Up**				
ENSOART00000025181	710.203	156.937	2.178	0.000
MSTRG.15961.3	16.18	6.473	1.322	0.000
ENSOART00000020275	7.803	2.347	1.733	0.000
ENSOART00000006297	6.720	1.347	2.319	0.000
ENSOART00000005653	6.470	1.303	2.312	0.000
ENSOART00000022599	27.050	0.053	8.986	0.000
MSTRG.5729.1	12.727	0.000	16.958	0.000
ENSOART00000023302	1143.227	249.343	2.197	0.000
ENSOART00000015307	23.017	10.730	1.101	0.000
ENSOART00000010885	32.420	9.277	1.805	0.000
ENSOART00000024708	253.550	57.057	2.152	0.000
ENSOART00000024789	55.240	21.610	1.354	0.000
MSTRG.81402.16	9.087	4.413	1.042	0.000
ENSOART00000020829	7.807	1.093	2.836	0.000
ENSOART00000025702	237.103	52.200	2.183	0.000
ENSOART00000008854	12.180	1.507	3.527	0.000
ENSOART00000014592	10.040	0.317	4.987	0.000
ENSOART00000026678	302.540	75.643	2.000	0.000
ENSOART00000005585	8.677	0.003	11.346	0.000
ENSOART00000008244	5.387	0.070	6.266	0.000
ENSOART00000026731	755.063	139.233	2.439	0.000
ENSOART00000025387	1455.807	325.817	2.160	0.000
ENSOART00000000035	7377.263	3195.677	1.207	0.000
ENSOART00000014259	20.997	3.507	2.582	0.000
MSTRG.8377.16	5.150	2.413	1.094	0.000
ENSOART00000010169	147.077	30.703	2.260	0.000
ENSOART00000025595	82.747	7.800	3.407	0.000
MSTRG.81610.1	12.317	0.600	4.360	0.000
ENSOART00000024451	1050.853	235.157	2.160	0.000
ENSOART00000015510	7.787	0.740	3.395	0.000
ENSOART00000026969	865.360	229.537	1.915	0.000
ENSOART00000019042	5.793	0.107	5.763	0.000
MSTRG.39618.2	26.370	0.767	5.104	0.000
ENSOART00000024324	120.010	52.420	1.195	0.000
ENSOART00000025987	2128.780	489.940	2.119	0.000
ENSOART00000026461	973.247	219.280	2.150	0.000
ENSOART00000002985	5.157	0.833	2.629	0.000
ENSOART00000016419	6.227	0.083	6.223	0.000
ENSOART00000024392	60.470	15.287	1.984	0.000

ENSOART00000000924	36.477	17.227	1.082	0.000
ENSOART00000025852	160.717	38.717	2.068	0.000
MSTRG.61223.7	11.623	2.093	2.473	0.000
ENSOART00000026632	1136.277	252.220	2.171	0.000
ENSOART00000026983	63.547	7.383	3.105	0.000
ENSOART00000026987	1313.017	298.130	2.139	00.000
ENSOART00000007926	5.960	0.040	7.219	0.000
**Down**				
ENSOART00000018278	4.330	28.510	-2.719	0.000
ENSOART00000003532	14.210	56.950	-2.003	0.000
ENSOART00000008431	10.480	38.843	-1.890	0.000
ENSOART00000006058	5.027	35.453	-2.818	0.000
ENSOART00000001148	4.457	11.123	-1.320	0.000
ENSOART00000009973	25.540	68.610	-1.426	0.000
ENSOART00000000477	256.930	945.687	-1.880	0.000
ENSOART00000020515	2.350	7.727	-1.717	0.000
ENSOART00000009316	55.837	158.033	-1.501	0.000
ENSOART00000008658	0.533	9.727	-4.189	0.000
ENSOART00000016952	6.433	26.850	-2.061	0.000
ENSOART00000027084	3.680	31.967	-3.119	0.000
ENSOART00000004802	12.380	47.540	-1.941	0.000
ENSOART00000006681	8.890	34.417	-1.953	0.000
ENSOART00000012169	7.340	19.843	-1.435	0.000
ENSOART00000016422	0.000	8.250	-16.332	0.000
MSTRG.36400.1	0.000	15.493	-17.241	0.000
ENSOART00000020009	4.600	26.227	-2.511	0.000
ENSOART00000020987	7.790	63.100	-3.018	0.000
ENSOART00000016107	3.317	27.070	-3.029	0.000
ENSOART00000000483	0.583	5.513	-3.241	0.000
ENSOART00000019853	11.340	32.723	-1.529	0.000
ENSOART00000017039	10.267	26.833	-1.386	0.000
ENSOART00000011132	2.133	43.113	-4.337	0.000
ENSOART00000001470	4.420	10.440	-1.240	0.000
ENSOART00000007036	0.877	22.787	-4.700	0.000
ENSOART00000021942	6.893	34.447	-2.321	0.000
MSTRG.87688.2	0.000	14.313	-17.127	0.000
ENSOART00000003197	25.767	75.417	-1.549	0.000
ENSOART00000001738	17.440	105.070	-2.591	0.000
ENSOART00000009914	17.233	94.600	-2.457	0.000
ENSOART00000014309	0.023	10.300	-8.786	0.000
ENSOART00000008853	0.000	21.480	-17.713	0.000
MSTRG.77996.2	2.250	16.420	-2.867	0.000
ENSOART00000010404	158.520	318.193	-1.005	0.000
MSTRG.37462.2	0.247	5.100	-4.370	0.000

ENSOART00000010059	0.530	19.603	-5.209	0.000
ENSOART00000002697	1.153	19.660	-4.091	0.000
ENSOART00000010960	7.627	25.670	-1.751	0.000
ENSOART00000018756	2.570	52.057	-4.340	0.000
MSTRG.56516.25	0.000	11.720	-16.839	0.000
ENSOART00000004211	21.830	48.247	-1.144	0.000
ENSOART00000005586	0.000	12.700	-16.955	0.000
ENSOART00000001136	40.817	86.867	-1.090	0.000
ENSOART00000008245	2.853	9.440	-1.726	0.000
ENSOART00000018233	4.537	15.943	-1.813	0.000
ENSOART00000011195	10.740	192.603	-4.165	0.000
ENSOART00000013061	2.363	13.543	-2.519	0.000
ENSOART00000018984	9.877	25.447	-1.365	0.000
ENSOART00000001186	37.857	131.397	-1.795	0.000
ENSOART00000011880	9.077	33.877	-1.900	0.000
ENSOART00000022712	65.873	422.400	-2.681	0.000
ENSOART00000022713	1.443	15.593	-3.433	0.000
ENSOART00000007695	12.270	33.660	-1.456	0.000
ENSOART00000013725	1.477	6.417	-2.119	0.000
ENSOART00000001229	1.330	9.853	-2.889	0.000
MSTRG.13431.6	0.180	24.907	-7.112	0.000
ENSOART00000000572	1.210	5.043	-2.059	0.000
MSTRG.33900.1	0.000	6.377	-15.961	0.000
ENSOART00000016036	2.633	27.947	-3.408	0.000
ENSOART00000021489	5.067	23.603	-2.220	0.000
ENSOART00000001151	0.460	5.723	-3.637	0.000
MSTRG.87994.1	0.000	5.840	-15.834	0.000
ENSOART00000015928	6.190	31.717	-2.357	0.000
ENSOART00000005170	8.800	27.230	-1.630	0.000
ENSOART00000019044	0.000	11.360	-16.794	0.000
ENSOART00000001051	5.793	43.483	-2.073	0.000
ENSOART00000013591	10.333	45..587	-1.906	0.000
ENSOART00000014234	0.043	37.867	-9.771	0.000
ENSOART00000013492	5.220	19.147	-1.875	0.000
ENSOART00000013416	0.000	9.793	-16.580	0.000
MSTRG.4392.1	0.130	37.043	-8.155	0.000
ENSOART00000004239	153.953	323.310	-1.070	0.000
ENSOART00000008811	0.227	5.767	-4.669	0.000
MSTRG.15917.4	1.750	15.507	-3.147	0.000
ENSOART00000008562	4.933	22.730	-2.204	0.000
MSTRG.16260.13	0.000	5.653	-15.787	0.000
MSTRG.73785.3	1.920	11.957	-2.639	0.000
ENSOART00000003731	11.977	33.320	-1.476	0.000
ENSOART00000021938	7.383	52.033	-2.817	0.000

ENSOART00000020258	20.943	141.280	-2.754	0.000
ENSOART00000018694	13.190	35.107	-1.412	0.000
ENSOART00000016681	0.430	6.437	-3.904	0.000
ENSOART00000013884	0.037	73.297	-10.965	0.000

[1]Transcript ID：Transcript number.

Supplementary Table S7. Effect of GAS on the morphology of stomachs in fattening sheep.

**Position**	**Item**	**CON (um)**	**GAS (um)**
Rumen	Muscularis	1010.62±34.26	895.97±33.10
Reticulum	Muscularis	1335.28±76.93	1251.4±69.39
Omasum	Muscularis	706.78±10.17	669.8±43.91
Abomasum	Muscularis	451.64±21.53	422.25±4.12

[a,b] Values within a row with different superscripts differ significantly at $P < 0.05$.

Supplementary Table S8. Effect of PRB on the morphology of intestinal segment in fattening sheep.

Position	Item	CON (um)	GAS (um)
Duodenum	Muscularis	431.17±3.29	503.87±49.57
	Villus	383.11±5.00	370.40±16.25
Jejunum	Muscularis	272.98±5.24	204.87±40.74
	Villus	362.08±38.72	348.11±22.62
Ileum	Muscularis	161.41±7.32	149.21±1.68
	Villus	276.27 ±4.31	239.41±14.98
Cecum	Muscularis mucosae	344.66±11.46	355.35±30.68
	Mucosa	396.35±14.37	327.92±36.14
Colon	Muscularis mucosae	276.68±12.00	319.39±14.81
	Mucosa	535.91±23.65	506.55±14.18
Rectum	Muscularis mucosae	363.97±8.92	393.86±7.60
	Mucosa	351.72±32.90	417.12±45.21

[a,b] Values within a row with different superscripts differ significantly at $P < 0.05$.

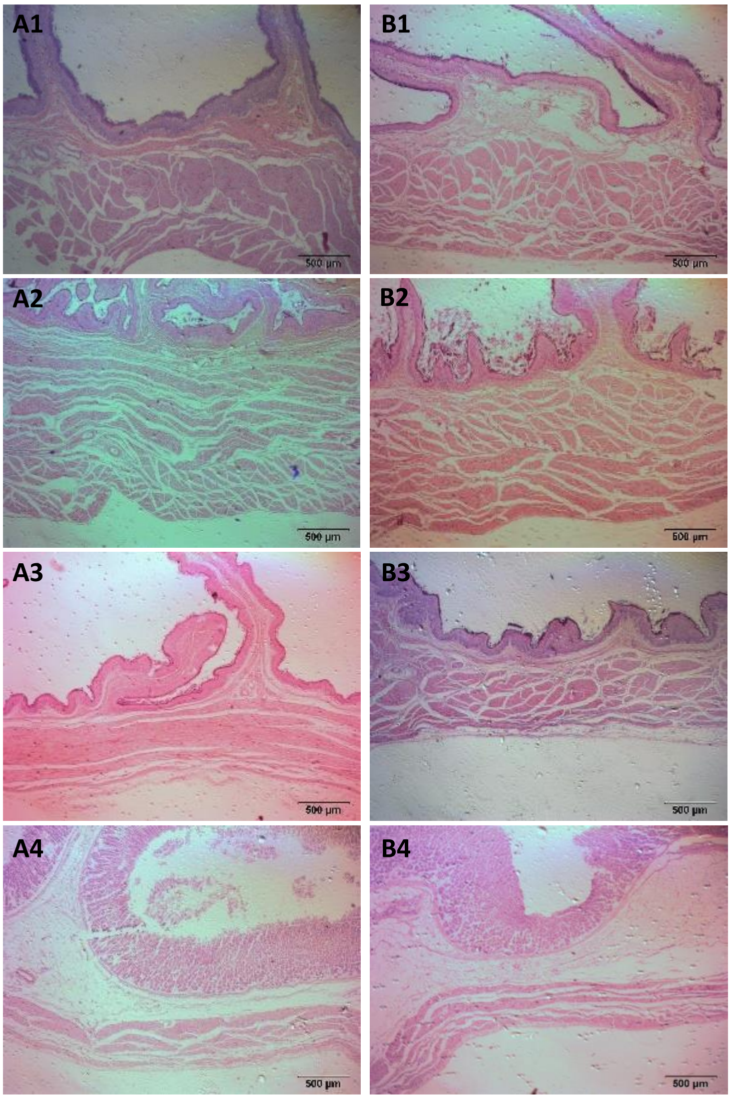

Supplementary Figure S1. Histomorphology of stomachs in fattening sheep fed with GAS diet (A) CON (B) GAS (1) Rumen (2) Reticulum (3) Omasum (4) Abomasum.

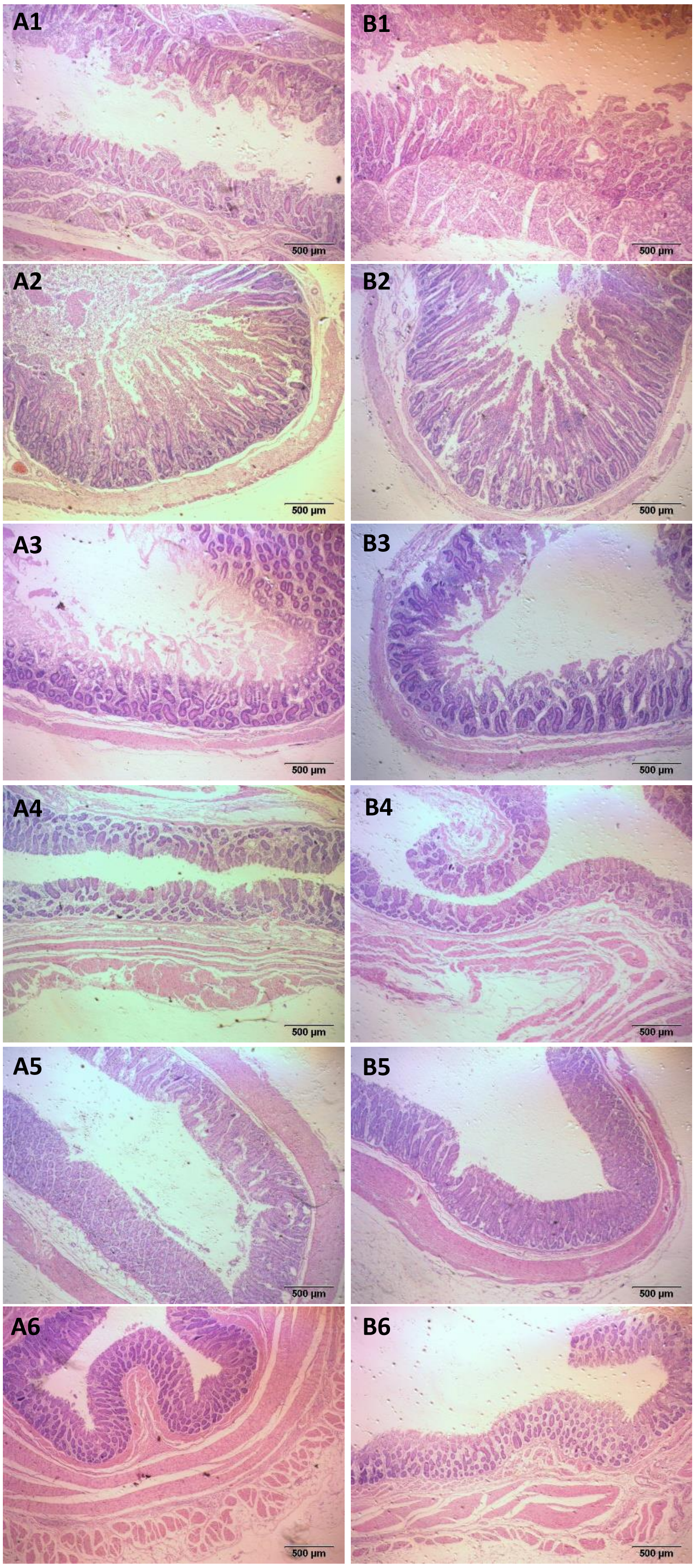

Supplementary Figure S2. Histomorphology of intestinal segments in fattening sheep fed with GAS diet (A) CON (B) GAS (1) Duodenum (2) Jejunum (3) Ileum (4) Cecum (5) Colon (6) Rectum.
